# Rapid Transient Transcriptional Adaptation to Hypergravity in Jurkat T Cells Revealed by Comparative Analysis of Microarray and RNA-Seq Data

**DOI:** 10.3390/ijms22168451

**Published:** 2021-08-06

**Authors:** Christian Vahlensieck, Cora S. Thiel, Jan Adelmann, Beatrice A. Lauber, Jennifer Polzer, Oliver Ullrich

**Affiliations:** 1 Faculty of Medicine, Institute of Anatomy, University of Zurich, Winterthurerstrasse 190, 8057 Zurich, Switzerland; christian.vahlensieck@uzh.ch (C.V.); janade@student.ethz.ch (J.A.); beatrice.lauber@anatomy.uzh.ch (B.A.L.); jennifer.polzer@uzh.ch (J.P.); 2 Air Force Center, Innovation Cluster Space and Aviation (UZH Space Hub), University of Zurich, Überlandstrasse 271, 8600 Dübendorf, Switzerland; 3 Space Biotechnology, Department of Machine Design, Engineering Design and Product Development, Institute of Mechanical Engineering, Otto-Von-Guericke-University Magdeburg, Universitätsplatz 2, 39106 Magdeburg, Germany; 4 Space Medicine, Ernst-Abbe-Hochschule (EAH) Jena, Department of Industrial Engineering, Carl-Zeiss-Promenade 2, 07745 Jena, Germany; 5 Zurich Center for Integrative Human Physiology (ZIHP), University of Zurich, Winterthurerstrasse 190, 8057 Zurich, Switzerland; 6 Kennedy Space Center (KSC), Space Life Sciences Laboratory (SLSL), 505 Odyssey Way, Exploration Park, FL 32953, USA

**Keywords:** microgravity, hypergravity, altered gravity, spaceflight, ground-based facilities, microarray, RNA-Seq, transcriptomics, Jurkat T cells

## Abstract

Cellular responses to micro- and hypergravity are rapid and complex and appear within the first few seconds of exposure. Transcriptomic analyses are a valuable tool to analyze these genome-wide cellular alterations. For a better understanding of the cellular dynamics upon altered gravity exposure, it is important to compare different time points. However, since most of the experiments are designed as endpoint measurements, the combination of cross-experiment meta-studies is inevitable. Microarray and RNA-Seq analyses are two of the main methods to study transcriptomics. In the field of altered gravity research, both methods are frequently used. However, the generation of these data sets is difficult and time-consuming and therefore the number of available data sets in this research field is limited. In this study, we investigated the comparability of microarray and RNA-Seq data and applied the results to a comparison of the transcriptomics dynamics between the hypergravity conditions during two real flight platforms and a centrifuge experiment to identify temporal adaptation processes. We performed a comparative study on an Affymetrix HTA2.0 microarray and a paired-end RNA-Seq data set originating from the same Jurkat T cell RNA samples from a short-term hypergravity experiment. The overall agreeability was high, with better sensitivity of the RNA-Seq analysis. The microarray data set showed weaknesses on the level of single upregulated genes, likely due to its normalization approach. On an aggregated level of biotypes, chromosomal distribution, and gene sets, both technologies performed equally well. The microarray showed better performance on the detection of altered gravity-related splicing events. We found that all initially altered transcripts fully adapted after 15 min to hypergravity and concluded that the altered gene expression response to hypergravity is transient and fully reversible. Based on the combined multiple-platform meta-analysis, we could demonstrate rapid transcriptional adaptation to hypergravity, the differential expression of the ATPase subunits ATP6V1A and ATP6V1D, and the cluster of differentiation (CD) molecules CD1E, CD2AP, CD46, CD47, CD53, CD69, CD96, CD164, and CD226 in hypergravity. We could experimentally demonstrate that it is possible to develop methodological evidence for the meta-analysis of individual data.

## 1. Introduction

Gravity has been a constant presence for billions of years, throughout the Earth’s history [1,2]. All life as we know it has developed under the constant influence of Earth’s gravitational field and has adapted on all structural levels to it [3]. Surprisingly, since the early days of spaceflight, it has been observed that the immune system deteriorates during and after exposure to spaceflight conditions [4,5,6]. Next to straightforward drivers including radiation, altered gravity (hyper- and microgravity) is a key factor behind the effects on the level of the immune system down to isolated cells [7,8]. Generally, several cell types have been observed to be influenced by altered gravity, but immune cells belong to the most affected (reviewed in [9,10,11]). More recently, it was observed that cellular reactions to altered gravity appear after seconds to minutes of exposure and include a plethora of effects, including reduced oxidative burst in microgravity and increased burst in hypergravity in macrophages [12,13], chromatin regulatory effects in microgravity [14], cell cycle regulation in microgravity [15], micro-RNA expression in simulated microgravity [16], altered immune cascade-associated messenger protein levels in hypergravity, and microgravity in T cells including surface concentrations of IL-2R and LAT [17], by significantly altering the expression of thousands of transcripts within 20 s of microgravity and hypergravity, and by almost fully adapting the transcriptome pool after 5 min of microgravity compared to 20 s [18,19,20] (reviewed in [21]).

Despite extensive research efforts, no underlying mechanism has been identified yet. Gravity itself is potentially too weak to be sensed directly [22,23]. There are hypotheses about the involvement of cytoskeletal structures and ion channels [24], but so far, no consistent theory has been experimentally confirmed. Therefore, gravitational sensing in immune cells requires further studies to pinpoint potentially involved mechanisms. One major approach is genome-wide transcriptomics: it shows initial effects towards hyper- and microgravity already after 20 s [18,19], is relatively easy to implement, and yields a high information density of results. Therefore, it is an ideal tool to study cellular effects. The transcriptomics techniques have rapidly developed in the last few years. After initial approaches characterizing the transcriptome with Sanger sequencing-based SAGE (Serial Analysis of Gene Expression) analysis starting in 1995 [25], the field gained traction upon the development of high-throughput methods, including microarray and RNA-Seq analyses [26]. Transcriptome studies are always endpoint measurements. This means that only one point in time is represented. In order to display cellular reactions and responses as a time course, the data from several studies must be combined. Especially in microgravity and hypergravity research, the generation of data sets is cumbersome and time-consuming. Therefore, it is immensely important that data sets from different microgravity platforms at different time points and from different research teams can be analyzed in meta-studies. Due to the enormous specialization, and the different biological model systems and research platforms, the question of methods and evidence of comparability of studies is crucial to create real knowledge from single data with the help of correct meta-analyses.

The NASA GeneLab database provides open access to different multi-omics data sets [27,28]. A NASA GeneLab database search revealed that many transcriptomics samples from successful altered gravity campaigns were analyzed on microarrays. However, more and more recently uploaded data sets are based on RNA-Seq analyses. To be able to fully leverage past altered gravity transcriptomics data, cross-data-set studies would likely include directly comparing microarray and RNA-Seq data. The two techniques rely on very different approaches; therefore, they have their pros and cons [29,30,31,32]. Although RNA-Seq is described to be more sensitive than microarray data, the agreeability between both platforms is high [33]. However, this effect is not fully generalizable since the concordance highly depends on the type of treatment in differential gene expression studies [34].

In our previous studies, we were able to show a high degree of adaptation on the transcriptional level after short-term hyper- and microgravity exposure and we concluded that human immune cells are equipped with a robust and efficient adaptation potential when challenged with altered gravitational environments [18,20]. We further analyzed the reproducibility of hypergravity exposure during a suborbital rocket flight by centrifugation at 9× *g* in a ground centrifuge [35]. In the current study, we complemented our existing microarray data sets of 20 s and 75 s of hypergravity exposure with an additional short-term 15 min time point and investigated the dynamics of gene expression with respect to cellular transcriptional adaptation processes. High accelerations occurred not only in cell experiments during the flight profile of suborbital ballistic rockets, but also in the bloodstream of the arterial system [36,37] and during routine laboratory protocols for cell culture and cell separation. We were able to identify that differential gene expression for hypergravity conditions is mostly transient. The majority of transcripts either showed no response or were upregulated after 20 s of hypergravity. These upregulated transcripts subsequently adapted to 1× *g* levels or were even downregulated after 75 s or 15 min.

Finally, we directly compared microarray and RNA-Seq analyses for one of our data sets, to evaluate whether these two different analysis techniques could be used in combined meta-study investigations.

To the best of our knowledge, large transcriptomic data sets have only been compared for RNA-Seq and microarray analyses for research studies not associated with altered gravity [38,39,40,41]. The good overall agreement of our microarray and RNA-Seq data allowed us to include two further long-term data sets available in the NASA GeneLab database into our combined analysis. We could show long-term transcriptional effects for the previously described gravi-sensitive vacuolar H+-ATPase (V-ATPase) ATP6V1 as well as for several Cluster of Differentiation cell adhesion molecules (CD), surface proteins that are highly involved in immune system functionality.

## 2. Results

In our previous transcriptomics analyses, we detected high temporal dynamics of gene expression under short-term hyper- and microgravity. By comparing two time points, (i) 20 s and (ii) 75 s, we could document fast adaptation responses in altered gravity, namely hypergravity for human Jurkat T cells. The aim of the current study was to investigate whether the transcriptome response is rapidly adaptive, continuous, or transient. For this reason, we chose the time point of 15 min of hypergravity exposure.

**Figure 1:**
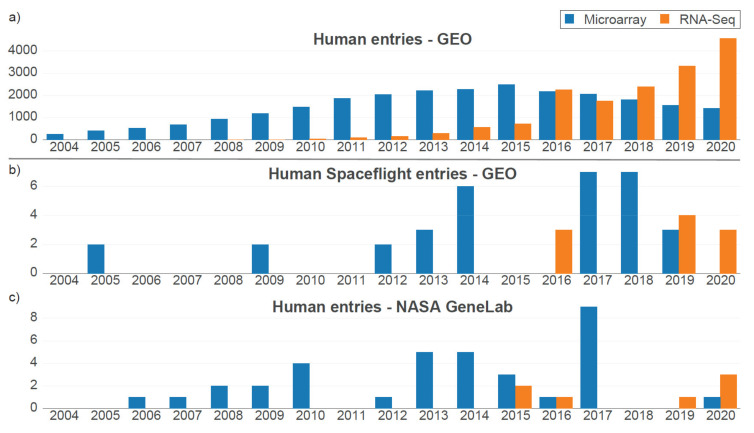
Deposition of RNA-Seq and transcriptomics microarray data sets over time. (**a**) Human transcriptomics data sets deposited in the Gene Expression Omnibus database per year. For most journals, data sets must be made available upon publication. Therefore, deposited data sets directly represent which technologies were used in publications in each year. A general shift towards RNA-Seq appeared in the year 2016. (**b**) Subset of spaceflight-related human transcriptomics data sets deposited in the Gene Expression Omnibus database. The much smaller subset displays the higher popularity of microarray technology compared to the overall set, with a clear trend towards RNA-Seq only in 2020. (**c**) All human entries in the spaceflight/space biology-specific NASA GeneLab database. There is a high correspondence to human spaceflight entries in the GEO database.

Furthermore, we performed a database search to identify other publicly available data sets based on the same or a similar cell type and to obtain an overview of the predominant techniques used for gene expression analyses. We filtered entries in the NCBI Gene Ontology Omnibus (GEO) database for the general field of human transcriptomics and discovered a peak usage of microarrays in the year 2015 and a breakeven with RNA-Seq data sets in the year 2016 (Figure 1a). For human spaceflight data sets, however, both the filtered GEO database and the specific NASA GeneLab database showed a dominating usage of microarrays after 2016, with only a small cumulative lead for RNA-Seq data sets (Figure 1b,c). Only in 2020, RNA-Seq data set depositions clearly dominated in the field of spaceflight and altered gravity. Given the fact that both methodologies are used and that there will be most likely a shift towards RNA-Seq analyses in the future also in the field of gravitational biology, we also wanted to evaluate the comparability of these two technologies. In particular, cross-validation meta-studies would likely have to implement microarray and RNA-Seq data. We therefore compared the different transcriptomics technologies on a hypergravity sample set that was generated on a pipette centrifuge rotating for 15 min at 9× *g* to be able to directly characterize the strengths and potential weaknesses of RNA microarray and RNA-Seq for our short-term altered gravity samples.

The same RNA from the hypergravity samples and additional 1× *g* control samples was analyzed on Affymetrix GeneChip Human Transcriptome Array 2.0 microarrays and in parallel with a standard Illumina RNA-Seq library with 25 million reads per sample and 75 bp paired end reads. The microarray, a recent development of Affymetrix, is not only able to screen 44,699 transcript clusters but additionally has 339,146 probes against splicing junctions, which allows for the specific detection of splicing sites. Therefore, it is a highly standardized, mature representative of microarray development [42]. The RNA-Seq data set was generated in a standard approach with a sequencing depth of 25 million and 75 bp paired-end reads.

### 2.1. Comparison of RNA Microarray versus RNA-Seq Overall Distribution and Differential Gene Expression

Before the comparative analysis between short-term hypergravity, 15 min of hypergravity, and external microarray and RNA-Seq data sets, we assessed the comparability between microarray and RNA-Seq transcriptomic data sets (Supplementary Figures S1–S3, Supplementary Results and Discussion). As previously discussed, the comparability between these two transcriptomics platforms heavily depends on the structure of differential gene expression [34]. The RNA-Seq data set had a higher dynamic range of fold change distributions. The microarray distribution was shifted and narrowed compared to the RNA-Set distribution, which had a tail of downregulated genes. Generally, the microarray detected only 2511 differentially expressed genes (DEGs, defined as False Discovery Rate FDR <0.05), in contrast to 5074 for RNA-Seq; in particular, less upregulated genes were detected by the microarray. The DEGs detected by RNA-Seq resembled two normal distributions; for the microarray, there was only one. These differences in appearance could be explained by the Robust Multiarray Averaging (RMA) normalization performed by microarray data analysis, which narrowed and shifted distributions, which was also the case when applying it to the RNA-Seq data set.

Of the 2511 DEGs, 60% could also be detected in the RNA-Seq set. However, this overlap was mostly driven by downregulated genes: 85% of the downregulated genes could also be detected in the RNA-Seq data set, in contrast to 3% (21 genes) for upregulated genes. The number of contradictory results (upregulated for one, downregulated for the other technology) was very low, i.e., only two DEGs; however, contradictory results are possible in general. When analyzing the overlap at stricter false discovery rates, a core set of DEGs emerged that was highly overlapping between the technologies. Overall, 92% of highly significant DEGs for the microarray could be detected in the RNA-Seq data set. The RNA-Seq data set had only an overlap of 44%, indicating that RNA-Seq was more sensitive and was able to identify further significant DEGs that could not be detected by the microarray. In summary, the general agreeability between both technologies was high, with weaknesses for upregulated genes. We could detect significantly differentially expressed genes that could be validated by both technologies.

### 2.2. A Comparison of Both Data Sets on an Aggregated Level Revealed Good Overall Agreeability

**Figure 2:**
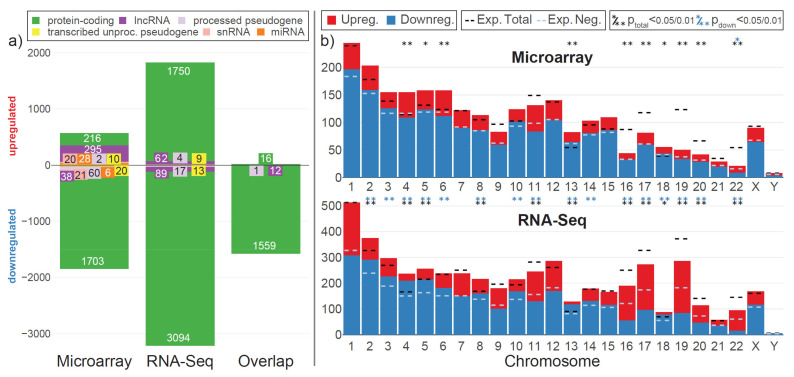
Characterization of differentially expressed genes on both technologies. (**a**) Ensemble gene biotypes of differentially expressed genes, split by up- and downregulation. On the horizontal axis, the three categories are: left—all differentially expressed genes (FDR-adjusted *p*-value < 0.05) for the microarray data set; middle—all differentially expressed genes (FDR-adjusted *p*-value < 0.05) for the RNA-Seq data set; right—the overlap that is significant for both technologies. Gene biotypes with less than 20 occurrences in the entire data set have been filtered out, leading to small deviations of absolute numbers compared to Supplementary Figure S3. (**b**) Chromosomal distribution of differential gene expression. Number of differentially upregulated (red) and downregulated (blue) genes per chromosome (X axis) for all three comparisons. The expected number (if there was no selection towards certain chromosomes) of total DEGs per chromosome (based on the fraction of differentially expressed genes for all genes and the number of detected genes per chromosome) is shown as a black dashed line; the expected number of downregulated genes out of up- and downregulated genes is shown as a dashed light blue line. Above each diagram, asterisks show if the actual number of all DEGs (upper row)/upregulated genes (lower row) lies significantly (*p* < 0.05: *, *p* < 0.01: **) above or below expectation.

As a next validation step, we wanted to compare the behavior of both data sets on an aggregated level. Hyper- and microgravity effects of the transcriptome usually consist of several thousand differentially expressed genes. Therefore, interesting parameters between the two technologies were the characterization of transcriptional response, clustering, and set analysis performance. The gene biotypes of differentially expressing genes were highly overlapping for downregulated genes (Figure 2a): for the microarray, 1703 out of 1848 (92%) downregulated genes were protein-coding and 2% coded for lncRNA. For RNA-Seq, 3094 out of 3213 (96%) coded for proteins, and 2.7% represented lncRNA. The microarray data set contained additional pseudogenes, snRNA-coding genes, and miRNA-coding genes. The downregulated overlap contained 1559 protein-coding genes, 12 lncRNA genes, and 1 processed pseudogene. For upregulated genes, the situation was different: in addition to the skew in overall distribution (Supplementary Figure S1), the transcriptional response of the microarray contained many more lncRNA genes—295 vs. only 62 for RNA-Seq, which corresponded to 52% of all upregulated genes vs. 3.4% for RNA-Seq. The upregulated overlap was only 16 protein-coding genes. The small deviation from the numbers from Supplementary Figure S3 was a consequence of filtering out low count biotypes. The overall distribution of differentially expressing genes over all chromosomes was comparable (Figure 2b): for most chromosomes, the number of DEGs followed expectation (indicated by dashed black lines). Some chromosomes significantly deviated from expectation (indicated with */** if Fisher’s exact test has an FDR-corrected *p*-value below 0.05/0.01). Chromosomes 4, 5, 13, 16, 17, 18, 19, 20, and 22 were found to be significantly altered for both data sets. The RNA-Seq data set was more sensitive in this regard, labeling more chromosomes as deviating (except for chr 6), but the general trend towards more/less was conserved along different chromosomes. The number of up- vs. downregulated genes was slightly more different between data sets. For the microarray set, only chromosome 22 was indicated to have more upregulated genes than expected, and no chromosome had significantly more downregulated genes. For RNA-Seq, additionally to chr 22, chromosomes 2, 3, 4, 5, 6, 8, 10, 11, 13, 14, 16, 17, 18, 19, and 20 also had significantly different ratios between upregulation and downregulation. Generally, for the microarray data set, the DEGs on the chromosomes were shifted more towards downregulation, with only a few upregulated genes; therefore, the sensitivity of the Fisher’s exact test was reduced. Consequently, the agreeability on an aggregated level was high and able to compensate for deviations from the single gene level.

### 2.3. Characterization of Splicing Events

**Figure 3:**
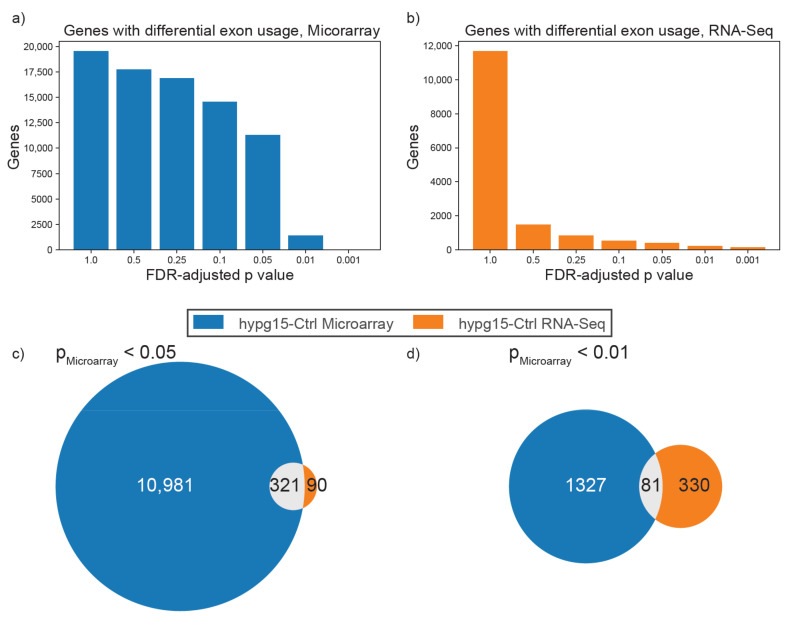
Differential exon usage (DEU)/alternative splicing detection for both technologies. (**a**) Visualization of the number of genes reported that have differential exon usage for the microarray data set at various FDR-adjusted *p*-value cutoffs. DEU detection was performed by the Affymetrix Transcriptome Analysis Console (TAC). (**b**) Visualization of how many genes are reported to have differential exon usage for the RNA-Seq data set. DEU detection was performed by the DEXSeq pipeline. (**c**,**d**) Overlap of detected DEU genes at FDR-adjusted *p*-value of 0.05 for RNA-Seq and 0.05/0.01 for microarray TAC. A list of all significant genes that overlap in 3c can be found in the supplement (Table S1).

Hyper- and microgravity have an effect on differential exon usage (DEU) and alternative splicing. We next tested if we could identify genes that were robustly found to be alternatively spliced for both technologies. The applied methods for the microarray and RNA-Seq technologies were very different: the specific microarray design with exon junction probes and splicing probes allowed for a direct quantification of exon events. For this analysis, the manufacturer-specific software “Transcriptome Analysis Console” (TAC) was used. For RNA-Seq, detection of differential exon usage relied on the subset of reads that randomly spanned an exon–exon junction. If such a read was detected, the associated transcript could either bear or lack an additional exon at its alignment site, depending on the read sequence. This application was not specifically engineered for DEU detection and therefore identified only a small fraction of the reads. Here, the implementation in the package DEXSeq was utilized. When filtering for an FDR-adjusted *p*-value of 0.05 for DEU, the microarray detected 11,302 DEU genes while RNA-Seq only detected 411 DEU genes (Figure 3a,b). When overlapping these, 321 of 411 (78%) of DEU genes for RNA-Seq were included in the microarray set at a cutoff of 0.05 (Figure 3c). The entire size of the data set for RNA-Seq was 13,719 genes. Since 11,302 DEU genes for the microarray was almost as large as the entire RNA-Seq data set, the significant overlap of DEU genes could also appear purely by chance. Therefore, we selected a stricter cutoff of *p* < 0.01 for the microarray set (Figure 3d): the overlap was significantly smaller, and only 81 out of 411 DEU genes for RNA-Seq were also present in the microarray set. These 81 genes can be found in the supplement (Table S1). We conclude that the specific design of the microarray allowed for much more sensitive detection of alternative splicing and differential exon usage, respectively.

### 2.4. Differential Gene Expression Shows High Agreeability between Previous Short-Term Hypergravity Data and the Current Fifteen-Minute Study

**Figure 4:**
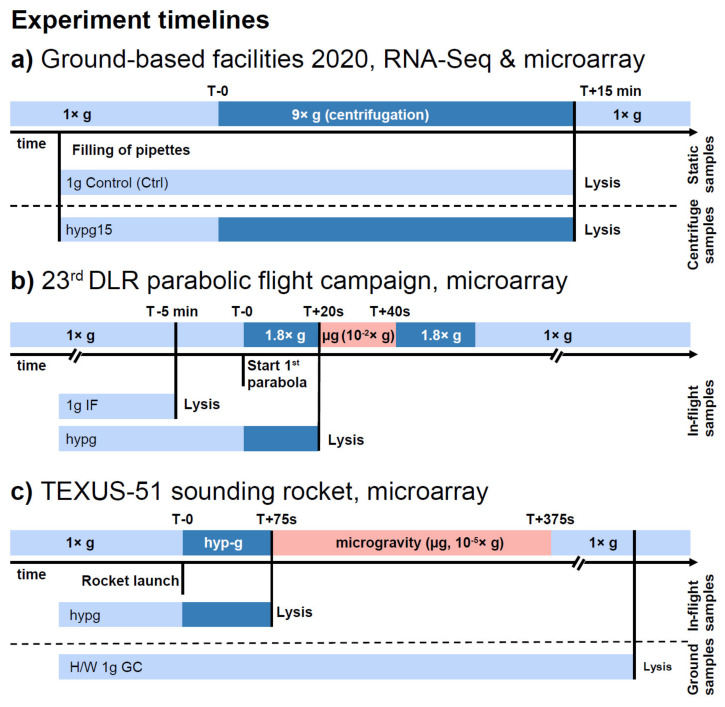
Experiment timelines of all data sets utilized in the short-term hypergravity cross-data-set study. (**a**) Ground-based facility study investigating the differential gene expression in human Jurkat T cells after 15 min of hypergravity exposure at 9× *g*. The same set of samples was analyzed by RNA-Seq and microarray and the results of the two technologies were compared. (**b**) During the 23rd DLR parabolic flight campaign, human Jurkat T cells were exposed to 20 s of hypergravity and 20 s of microgravity, respectively. The differential gene expression was analyzed by microarray technology. (**c**) During the TEXUS-51 sounding rocket campaign, human Jurkat T cells were exposed to 75 s of hypergravity and 5 min of microgravity. Transcriptomics analyses were performed by microarray technology.

**Figure 5:**
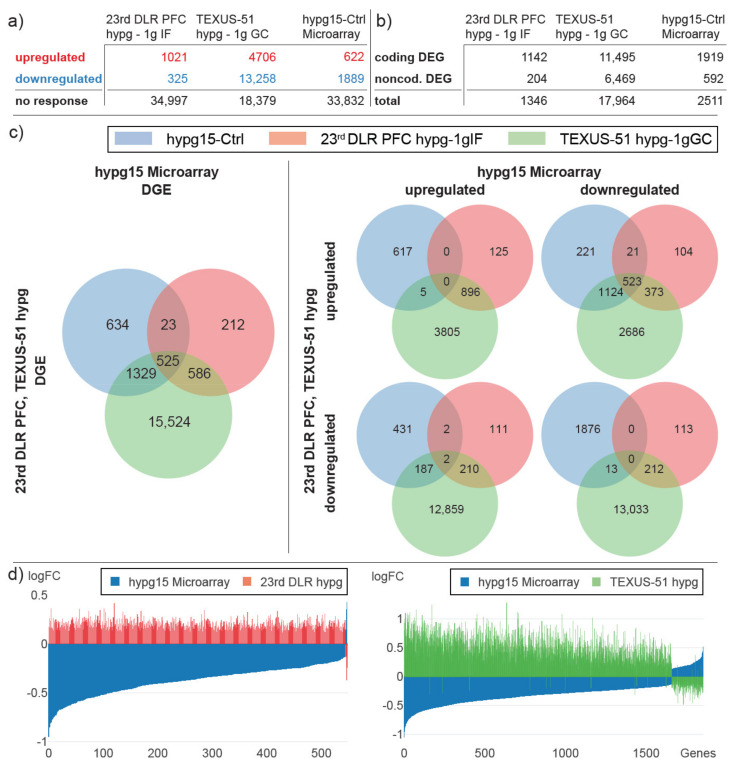
Cross-comparison of transcriptional behavior with short hypergravity microarray transcriptomics data sets, 23rd DLR PFC 20 s 1.8× *g* hypergravity (hypg) vs. 1× *g* in flight control (1gIF) and TEXUS-51 75 s ~9× *g* baseline hypergravity (hypg) vs. 1× *g* ground control (1gGC). 15 min hypergravity, microarray data only. (**a**) Significantly up- and downregulated genes and non-responsive genes for the two previous data sets and the hypg15 data set from this study, microarray data set only. (**b**) Significantly differential expressed genes, aggregated by protein-coding genes and noncoding genes for all three data sets. (**c**) Overlap between differentially expressed genes for all three data sets. Left: all significantly differentially expressed genes. Right: differentially expressed genes, split by upregulated and downregulated genes for the hypg15 microarray data set (horizontal axis) and by upregulated and downregulated genes for both external data sets (vertical axis). (**d**) Corresponding fold changes of genes that are differentially expressed both in hypg15 and 23rd DLR PFC hypg (left), resp. TEXUS-51 hypg (right). For each gene, two fold changes are displayed by two differentially colored bars at the same location on the horizontal axis.

Based on the high overall agreeability between the RNA-Seq data set and the microarray data set (Figure 4a), our next aim was to compare the data to two previous studies from our lab, 20 s of 1.8× *g* hypergravity during the 23rd DLR parabolic flight campaign (Figure 4b) and 75 s of hypergravity (with a median gravity of approximately 9× *g* and a peak of 12–13× *g*) during the TEXUS-51 sounding rocket campaign (Figure 4c). Firstly, we compared the two previous data sets to the hypg15-Ctrl (comparison between 15 min of 9× *g* hypergravity samples versus 1× *g* control samples) microarray data set only, since all were recorded on Affymetrix HTA2.0 microarrays (Figure 5). The number of up- and downregulated genes was the smallest for the 20 s data set and largest for the 75 s data set, with 17,964 differentially expressed genes (Figure 5a). After 20 s of hypergravity, upregulated genes dominated, with 76% of all DEGs; after 75 s, this shifted to downregulated genes, with 74% of all DEGs, and remained in this range after 15 min at 75%. At all points in time, far more protein-coding genes were differentially expressed than noncoding genes (Figure 5b); however, the ratio changed from 85% after 20 s over 64% after 75 s to 76% after 15 min.

The overlap between all three data sets was high (Figure 5c), with 525 DEGs that appeared in all three data sets and an additional 1329 only between TEXUS-51 hypg-Ctrl and hypg15-Ctrl. However, when separating by up- and downregulation of DEGs in the hypg15 data set and in the two previous short-term data sets (Figure 5c right side), the large overlap was almost uniquely present for opposite directions, with only 18 genes that were regulated in the same direction between hypg15-Ctrl and TEXUS-51 hypg and none for the 20 s data set. This counter-response could be further illustrated by plotting the fold changes of all genes that were differentially expressed in both the hypg15-Ctrl data set and one of the previous short-term data sets (Figure 5d).

**Figure 6:**
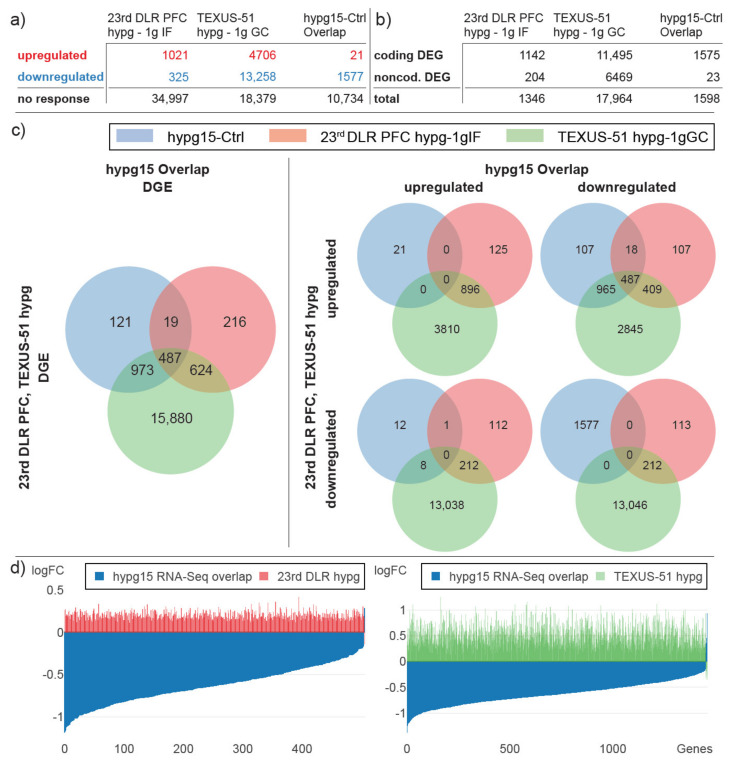
Cross-comparison of transcriptional behavior with short hypergravity microarray transcriptomics data sets, 23rd DLR PFC 20 s 1.8× *g* hypergravity (hypg) vs. 1× *g* in flight control (1gIF) and TEXUS-51 75 s ~9× *g* baseline hypergravity (hypg) vs. 1× *g* ground control (1gGC), parallel to Figure 5. 15 min hypergravity, only genes that are differentially expressed in the microarray and RNA-Seq data set. (**a**) Significantly up- and downregulated genes and non-responsive genes for the two previous data sets and the hypg15 data set from this study, overlap between microarray and RNA-Seq data. (**b**) Significantly differential expressed genes, aggregated by protein-coding genes and noncoding genes for all three data sets. (**c**) Overlap between differentially expressed genes for all three data sets. Left: all significantly differentially expressed genes. Right: differentially expressed genes, split by upregulated and downregulated genes for the hypg15 overlap data set (horizontal axis) and by upregulated and downregulated genes for both external data sets (vertical axis). (**d**) Corresponding fold changes of genes that are differentially expressed both in hypg15 and 23rd DLR PFC hypg (left), resp. TEXUS-51 hypg (right). For each gene, two different fold changes are displayed by two differentially colored bars at the same location on the horizontal axis.

We then repeated the analysis between the two previous short-term data sets and the hypg15 data set but limited the analysis on the overlap that was cross-validated between both technologies for the hypg15 data set (Figure 6). Here, only genes that were present in both the RNA-Seq and the microarray data sets were included; DEGs were regulated in the same direction in both data sets. As expected, the number of differentially expressed genes was decreased for the hypg15 data set, with only 21 upregulated genes remaining (Figure 6a). Further, as already described in Figure 2, the overlap data set mostly reported coding DEGs (Figure 6b). Interestingly, the overlap of differentially expressed genes was still at 487 genes, which is 93% of the analysis based only on microarray data (Figure 6c). This highly agreeable behavior was also reflected in the fold change distribution (Figure 6d). Therefore, the general agreeability between the previous short-term hypergravity data sets and the current 15 min data set was still present when only comparing DEGs that could be confirmed by both technologies.

### 2.5. Transient Transcriptional Response between Twenty-Second and Fifteen-Minute Hypergravity

**Figure 7:**
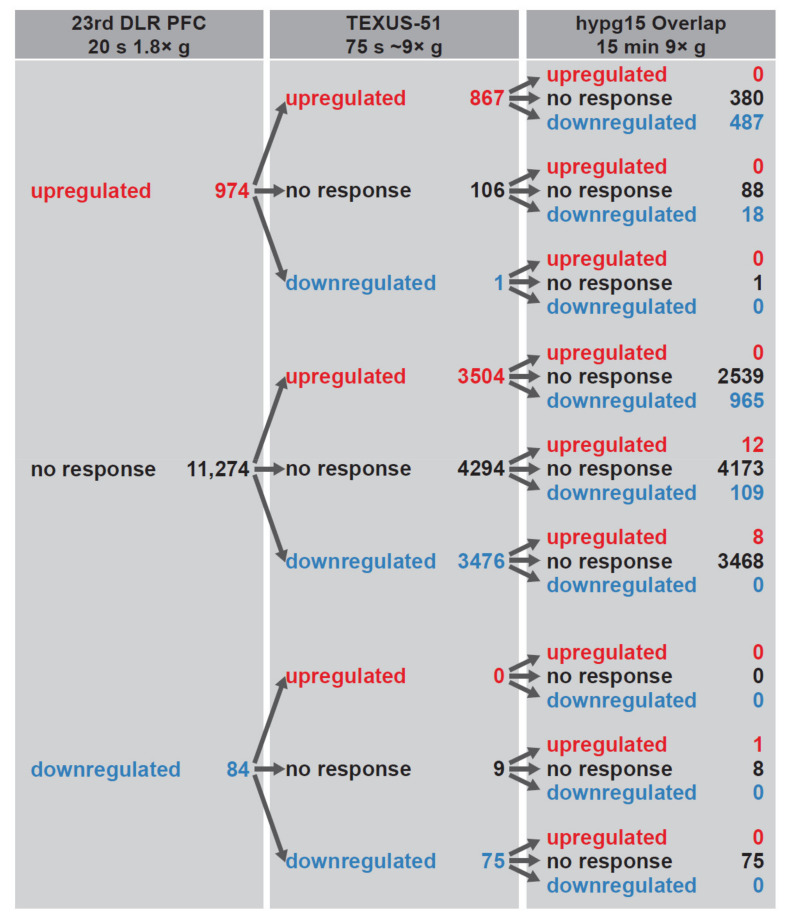
Corresponding differential expression between the 20 s 1.8× *g* 23rd DLR PFC hypg vs. 1× *g* IF data set, the 75 s ~9× *g* TEXUS-51 hypg-1gGC data set, and the 15 min hypg15 vs. Ctrl data set. For the first data set, genes were separated into those that were significantly upregulated, downregulated, and not differentially expressed. Then, the genes in every group were split into those that were differentially upregulated, downregulated, or not differentially expressed in the TEXUS-51 comparison, resulting in 9 categories. These 9 categories were consecutively split into 27 categories, depending on the behavior of the genes in the hypg15 vs. Ctrl microarray data set.

Because overlap analysis between differentially expressed genes of 20 s, 75 s, and 15 min of hypergravity revealed rapid and opposite transcription effects, we therefore analyzed the temporal dynamics of transcriptional regulation. We separated all genes into three categories: (1) upregulated after 20 s of hypergravity, (2) downregulated, and (3) genes that did not show a response (Figure 7). These three subsets were then further split into those that were upregulated after 75 s of hypergravity, downregulated, and non-responsive, resulting in nine subgroups. These were consecutively further split based on their behavior in the microarray/RNA-Seq overlap set after 15 min of hypergravity. We also performed the analysis based only on the microarray data sets (Supplementary Figure S4).

Interestingly, high continuity could be observed between the 20 s and the 75 s data set, with 89% genes upregulated after 75 s that were already upregulated after 20 s (Figure 7). Further, a large group of 3504 upregulated genes emerged after 75 s that were not upregulated after 20 s. The same effect was observed for downregulated genes (89% still downregulated genes and 3476 additional downregulated genes after 75 s); however, the group of initially downregulated genes was small, with only 84 genes. Surprisingly, genes that were upregulated after 75 s either showed no response (67%) or were downregulated (33%) after 15 min of hypergravity but were not upregulated anymore. Genes that were downregulated after 75 s did almost exclusively not respond anymore (99.8% of genes, Figure 7). The same effects could also be detected for the microarray-only analysis (Supplementary Figure S4); therefore, the behavior was not an analysis-based artifact of the small consensus overlap of upregulated genes in the two hypg15 data sets. We found that, out of the 1058 altered transcripts after 20 s hypergravity exposure, 116 (10.9%) adapted after 75 s and 1058 (100%) after 15 min. Out of the 7923 altered transcripts after 75 s hypergravity exposure, 7923 (100%) adapted after 15 min. We conclude that the initially altered transcriptional response to hypergravity is fully adapted after 15 min. Further, we conclude that the altered gene expression response immediately after alteration of the gravity environment is transient and fully reversible.

### 2.6. Transcriptome Adaptation to Hypergravity

**Figure 8:**
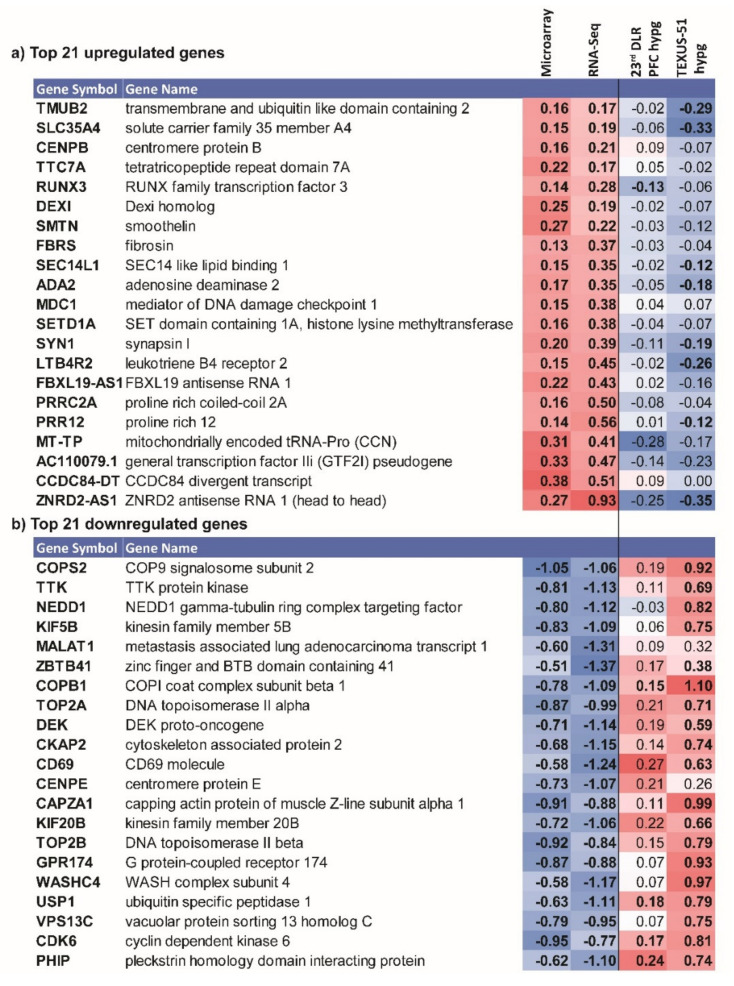
Overlapping genes from Supplementary Figure S3. (**a**) The 21 genes that are significantly upregulated for the microarray and the RNA-Seq data sets are listed. Additionally, the fold changes from two previous short-term hypergravity sets, 23rd DLR PFC hypg and TEXUS-51 hypg, are displayed. Significant fold changes are printed in bold. (**b**) The analysis from (**a**) for the 21 significantly downregulated genes with highest average fold changes for both data sets.

Next, the differentially expressed genes that were robustly detected for both platforms (called the consensus set) were characterized (Figure 8). The 21 upregulated genes (Figure 8a) and the top 21 downregulated genes with the strongest average fold change (Figure 8b) were listed. For these genes, we added the hypergravity versus 1× *g* comparison fold changes that had been detected for Jurkat T cells in the two previous flight missions: the 23rd DLR Parabolic Flight Campaign and the TEXUS-51 suborbital ballistic mission [18], both measured on Affymetrix HTA2.0 microarrays. Strikingly, the genes that were downregulated after 20 s and 75 s of hypergravity were upregulated after 15 min of hypergravity (Figure 8a). The same was true for the upregulated genes, which, after 15 min of hypergravity, were downregulated (Figure 8b). For the significant genes (bold font), this anticorrelation was true for all genes.

### 2.7. High Similarity in Gene Set Enrichment Analysis

**Figure 9:**
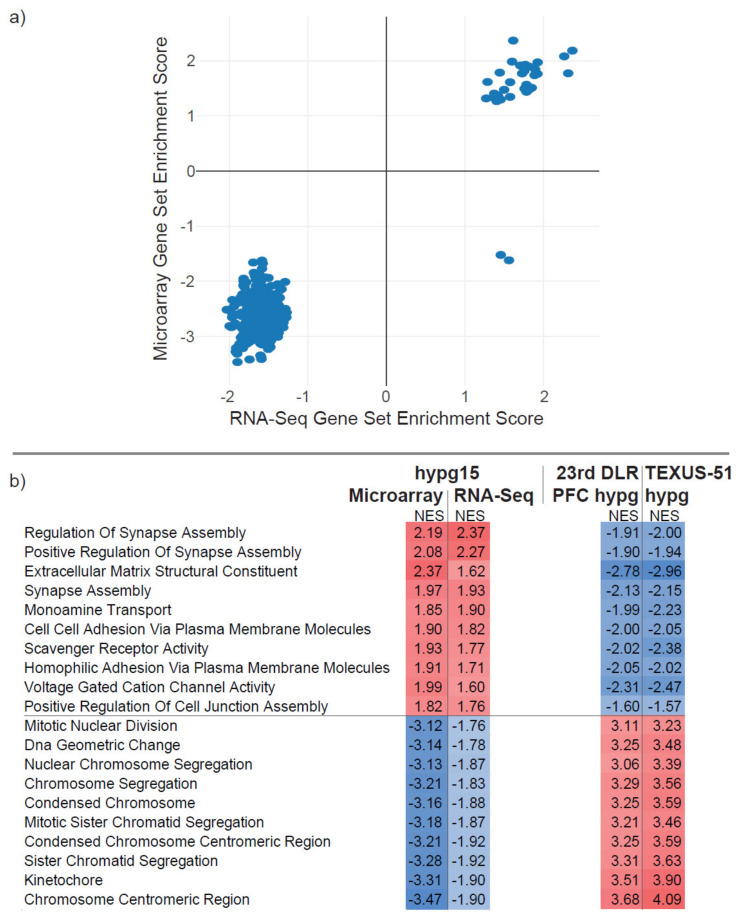
Gene set enrichment analysis. (**a**) Gene set enrichment analysis of all gene ontology gene sets for both technologies. Normalized enrichment scores (NES) for both data sets are plotted for all sets that showed significant enrichment in both technologies. The two contradictory outlier sets are “Regulation of small GTPase-mediated signal transduction” and “IRE1-mediated unfolded protein response”. (**b**) Top 10 gene sets with highest average positive NES and top 10 sets with highest average negative NES. The enrichment values for the two external hypergravity sets are given. All listed sets are significantly enriched for all experiments.

We wanted to test if the pattern of significantly altered genes could indicate any functional alterations. Therefore, a gene set enrichment analysis was our tool of choice, where the set of up- and downregulated DEGs was analyzed to determine whether they contained significantly more genes from a certain gene set than expected. Here, a quantitative gene set enrichment analysis was performed, testing for all gene ontology (GO) gene sets (Figure 9a,b). For both hypg15 data sets, significant gene sets could be identified. Further, we performed a cross-correlation with the hypergravity data sets from the 23rd DLR PFC and the TEXUS-51 campaign, representing 20 s and 75 s of hypergravity (compare Figure 4). Overall, many gene sets were identified as significantly enriched in both hypg15 analyses in parallel, with 31 overly upregulated and 275 overly downregulated sets (Figure 9a). The 10 significant gene sets with the highest average positive and highest average negative normalized enrichment score for both data sets are shown (Figure 9b), and they displayed high agreeability. All gene sets that were found to be significantly enriched for the two hypg15 data sets were also significantly enriched for the two previous sets. However, the normalized enrichment score (NES) values were inverted between the 20 s/75 s sets and the hypg15 sets, contrary to what happened for the hypg15 data sets. On the quantitative level, the normalized enrichment score, the measure of how much a gene set contains more significantly upregulated (positive NES) or downregulated (negative NES) genes than expected, was in good agreement between the hypg15 data sets (Figure 9a). Only two gene sets were significantly upregulated in the RNA-Seq data set and parallelly downregulated in the microarray data set: “Regulation of small GTPase-mediated signal transduction” and “IRE1-mediated unfolded protein response” (Figure 9a). The gene set with the highest and lowest NES for the hypg15 microarray showed the lowest and highest NES value for the two previous sets (Figure 9b). Interestingly, the 31 upregulated shared significant gene sets displayed high agreeability (Figure 9b). However, on the single gene level, there was only a small overlap for upregulated genes between the two data sets (compare Supplementary Figure S3). Generally, DEG characterization showed an excellent overlap of overall gene distribution, gene set enrichment, and partly biotype usage of downregulated genes.

### 2.8. A Cross-Correlation Analysis of Highly Validated Groups of Genes Revealed Temporal Consistency

**Figure 10:**
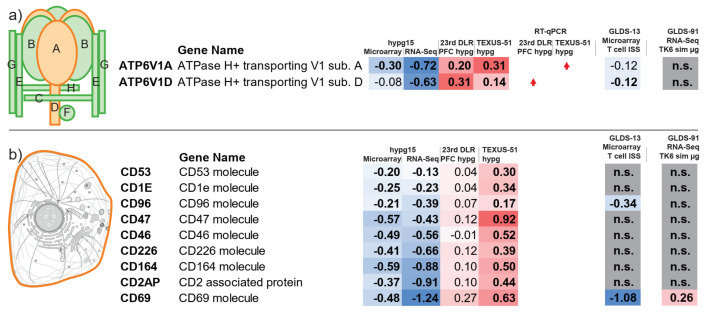
Highlighted groups of protein-coding genes have been analyzed for their behavior in different experiments separately. (**a**) ATP6V1 is the catalytic subcomplex of the ATP6V ion pump. The proteins ATP6V1A and ATP6V1D are known to be robustly affected by hypergravity. In the 23rd DLR PFC data set and the TEXUS-51 data set, they are upregulated under hypergravity, demonstrated by microarray data and independent RT-qPCR. Here, the fold changes for the hypg15 microarray and RNA-Seq data sets and the 23rd DLR PFC and the TEXUS-51 data sets are shown. Significant differential expressing genes (FDR < 0.05) are highlighted in bold. Two long-term external microgravity studies on immune cells were included (NASA GeneLab GLDS-13, 1.5 h microgravity onboard the ISS on activated human T cells, microarray data; NASA GeneLab GLDS-91, 48 h simulated microgravity (sim-µg) on TK6 immune cells, RNA-Seq) and the respective fold changes listed if significant (FDR < 0.05). (**b**) CD69 is among the strongest reacting genes for the hypg15 data sets. Here, all CD genes that are significantly differentially expressed (FDR < 0.05) in both hypg15 data sets are listed, including their fold changes in the microarray and RNA-Seq data set. Except for CD2AP and CD1E, all localize on the cell membrane, as indicated by the orange line. Two long-term external microgravity studies on immune cells were included (GLDS-13, 1.5 h microgravity onboard the ISS on activated human T cells, microarray data; GLDS-91, 48 h sim-µg on TK6 immune cells, RNA-Seq) and the respective fold changes listed if significant (FDR < 0.05).

We could show in a previous study that the proteins A and D from the V-type ATPase catalytic subunit (ATP6V1) are robustly upregulated in Jurkat T cells in short-term hypergravity [18]. This could be shown independently by microarray data and by real-time quantitative PCR (RT-qPCR) analysis. Therefore, we analyzed whether these genes were also robustly altered in our current data (Figure 10a, subunits are highlighted in orange). To further cross-validate these findings, we included two external reference sets from the NASA GeneLab [27] that we identified in our database search (Figure 1): GLDS-13 from T cells that were exposed to 3 days of microgravity and then either activated for 1.5 h in microgravity or 1.5 h in 1× *g* on a reference centrifuge onboard the ISS, measured on the microarray Affymetrix Human U133 Plus 2.0 [43]. Additionally, we included the data set GLDS-91, where TK6 lymphoblasts were exposed to 48 h simulated microgravity (sim-µg) in a HARV rotating suspension culture bioreactor, measured by RNA-Seq [44]. All experiments used here are listed in Table 1.

The two genes were known to be significantly upregulated under hypergravity in both previously generated short-term data sets 23rd DLR PFC and TEXUS-51 and showed increased RT-qPCR signal in one of each experiment (Figure 10a). For the hypg15 data sets, both units were significantly downregulated in RNA-Seq and the A unit significantly downregulated for the microarray (Figure 10a). Concerning the ISS data set GLDS-13, both genes were also reported to be significantly downregulated. For the sim-µg data set, the two genes were not reported to be significantly altered. Therefore, the initial upregulation effect turned into a downregulation effect after 15 min, which also appeared after 1.5 h of microgravity exposure, despite the fact that our experiment measured hypergravity and the ISS experiment measured microgravity. The data for four different time points and three different technologies pointed in the same direction in this regard.

Another interesting group of proteins were Cluster of Differentiation cell adhesion molecules (CD), surface proteins that are highly involved in immune system functionality [45], that also have shown specific behavior in altered gravity [46,47,48] and simulated microgravity [49]. One protein, CD69, was among the strongest downregulated genes (compare Figure 8b). We could identify nine CD proteins that showed significant consistent regulation (Figure 10b), which are listed in Table 2, including their chromosomal location, their subcellular localization, their Human Gene Database summary, and their presence on T cells. The genes were located on different chromosomes in different cytobands. We could not identify significant clustering on a certain cytoband. Except for CD2AP and CD1E, all were indicated to localize at the cell membrane or generally in membranes (Table 2, Figure 10b). All nine proteins were downregulated for both hypg15 data sets, but non-significantly upregulated (FDR > 0.05) for the PFC data set, and significantly upregulated for the TEXUS-51 data set. Interestingly, CD69 appeared significantly downregulated for the ISS data set and upregulated for the sim-µg data set. Additionally, CD96 also appeared downregulated for the ISS data set. Therefore, the initial upregulation after 20 and 75 s of CD molecules was followed by consecutive downregulation after 15 min, shown by two independent technologies. For CD69, the CD molecule with the strongest average downregulation after 15 min, the external GLDS-13 microgravity data set indicated significant downregulation after 1.5 h. For simulated microgravity on TK6 cells after 48 h, however, the gene appeared upregulated.

## 3. Discussion

Motivated by the observation of the altered immune responses of astronauts during mid- and long-term space missions, cellular reactions to microgravity have been studied on different levels for many decades [54]. More recently, it became evident that immune cells in in vitro culture display initial reactions to hyper- and microgravity after only a few seconds of exposure [12,13] (reviewed in [21]). Therefore, parabolic flight is a suitable platform for studying reactions in cellular systems and cell cultures including immune cells. It could be demonstrated on independent platforms and cell types that immune cells react with fundamental alterations of their transcription pool to short-term (20 s–5 min) microgravity and hypergravity [18,19]. These cellular reactions resemble a complex pattern of several hundreds or thousands of differentially expressed transcripts, which are not easy to interpret. A key approach to separating random fluctuations between experiments from true biological effects is cross-comparative studies between data sets from different campaigns [55,56]. Since time plays a crucial factor in these ultrashort reactions to hyper- and microgravity [35], cross-comparative studies investigating the effects on several timescales are an essential tool for further analyzing the mechanisms of cellular reactions to altered gravity. The fields of spaceflight and gravitational biology showed different ratios of usage of RNA-Seq compared to microarrays as transcriptomics platforms, with RNA-Seq becoming the dominating method from 2019 onwards (Figure 1). As mentioned above, one important tool for approaching complex mechanisms in biology is cross-experiment meta-studies [57,58]. Due to the relatively sparse data situation in the field, cross-comparability between data sets from different years can be a key point in experiment design. In this study, we compared microarray and RNA-Seq studies in order to enable cross-technology meta-studies in the future.

There have been various comparative studies between the two technologies. These include, but are not limited to, comparisons on the technical reproducibility [59], overall transcriptome distribution [26,60], applicability for T cell studies [41], usage in clinical endpoint predictions [40], or usage in dose–response studies [61]. Further, it already has been described that transcriptomics analysis on the level of gene sets instead of single genes is able to compensate for noise and other technical differences between the two technologies [61,62]. Generally, the agreeability between both technologies is described to be high [33]. However, this heavily depends on the internal structure of the transcriptomics data sets, e.g., how many genes are differentially expressed, how the fold changes are distributed, potential skew in up- and downregulation, etc. [34]. To the best of our knowledge, no comparison of large transcriptomic data sets for RNA-Seq and microarray analyses has been published in the field of gravitational biology so far.

Thus, we performed a comparative analysis of our internal data sets, where we analyzed the same short-term hypergravity samples with RNA-Seq and with Affymetrix HTA2.0 microarrays. For both types of analysis, we observed a skewed expression pattern (Supplementary Figure S1), being, most likely, an indicator of the non-equilibrium state of the cell. The microarray had some difficulties in representing this uneven distribution: the fold change distribution appeared more rectified, the overall distribution was shifted towards higher fold changes, and the overlap of significant genes with the RNA-Seq data set was only large for downregulated genes (Figure 2). Although the microarray analysis might have weaknesses for one direction of regulation, we could observe an overall good agreement between the two types of analyses for our data set. Most importantly, we were only able to detect two differentially expressed genes with contradictory fold changes that pointed in opposite directions for the two technologies. This means that there is virtually no risk of misidentifying upregulated genes as downregulated and vice versa.

Concerning differential exon usage and splicing the microarray, Affymetrix HTA2.0 showed better performance than the RNA-Seq data set (Figure 3). Due to its specific design with specialized junction probes, the HTA2.0 microarray was more sensitive to known splicing events. Specialized junction arrays are generally described to be more performant than RNA-Seq [63]. Detection of differential splicing for RNA-Seq, however, relies on sequencing reads that cover a splicing site by chance [64]. Therefore, the shorter the reads, the more unlikely the sequencing of a splice site is. The general performance, therefore, is inferior to the specialized microarray design.

On the level of gene biotypes, the two technologies showed similar performance (Figure 2a). Coding and noncoding genes could be identified in the upregulated and the downregulated fraction for both technologies, but to different extents. The microarray was able to identify more lncRNA genes and other non-protein-coding genes, especially in the upregulated fraction. This shift could be driven by signal strength: RNA-Seq requires a certain number of counts for a gene to be considered for differential expression analysis; additionally, genes with low counts have high variability. Microarrays, however, can be more sensitive for low-expressing genes due to their specific probe design, potentially also covering low-expressing noncoding genes [41]. The entire set of differentially expressing genes is similarly distributed on different chromosomes (Figure 2b). On the aggregated level of gene sets, the two technologies behaved very similarly, potentially compensating for differences that appeared on the level of single genes (Figure 8).

The overlap for downregulated genes was in very good agreement between both data sets. Moreover, we almost did not detect any contradictory overlaps, which would otherwise be a source of error. For global analyses such as chromosomal distributions, biotype usage, and gene set enrichment analyses, the microarray and RNA-Seq analyses behaved equally. Therefore, microarray and RNA-Seq data sets are a valuable resource for comparative studies and should be included in transcriptomics meta-studies whenever possible.

In the current study, we investigated a further time point additional to our existing transcriptomics data sets of 20 s and 75 s of hypergravity exposure to be able to detect the temporal dynamics of gene expression with respect to cellular transcriptional adaptation processes. The simulation of real flight profiles using laboratory centrifuges is difficult to achieve. For example, the complex acceleration profiles of suborbital ballistic missiles cannot be simulated with laboratory centrifuges. Moreover, acceleration profiles of real flight platforms consist of different hypergravity environments at different time points. Therefore, compromises must be made when defining the centrifuge parameters, and a hypergravity environment of 9× *g* was chosen as a compromise between the average acceleration (6× *g*) and the peak acceleration (approx. 12× *g*) of a suborbital ballistic rocket. Thus, no direct comparability of the data is possible, but the respective results in hypergravity were first compared with their respective control group of the respective platform and only afterwards compared with each other. However, the interpretation of the results is limited due to the heterogeneity of the hypergravity groups. Additionally, we wanted to assess if there is a major difference in analysis outcome if only microarray data sets or a combination of microarray and RNA-Seq data sets are used. We therefore exposed human Jurkat T cells to 15 min of hypergravity on a ground centrifuge and analyzed the same samples by microarray and RNA-Seq (Figure 2 and Figure 3). We then performed a cross-platform investigation including the previous 20 s and 75 s hypergravity microarray data sets and the newly generated 15 min hypergravity data set (Figure 5, Figure 6 and Figure 7). Despite the different hypergravity platforms, we were able to identify 525 overlapping DEGs for all three platforms. Most of them (523) were found in the intersection upregulated after 20 s and 75 s and downregulated after 15 min of hypergravity (Figure 5). The comparison of the 20 s and 75 s hypergravity data sets with the RNA-Seq/microarray overlapping DEGs showed highly similar results. In total, 487 overlapping DEGs were detected for all three hypergravity experiments, all of them being located in the intersection upregulated after 20 s and 75 s and downregulated after 15 min of hypergravity (Figure 6). This means that with the combination of RNA-Seq and microarray samples, we were able to identify 93% of the DEGs that were identified for the pure microarray analysis.

We were interested in the temporal dynamics of the differential gene expression of the investigated three time points and therefore grouped the analyzed genes into upregulated, downregulated, and non-responsive (Figure 7). We followed the different groups and identified the fate of the transcripts at each measured time point. After 20 s, most of the genes were either upregulated or non-responsive. The rapidly upregulated genes stayed after 75 s mainly upregulated. A minor fraction had already adapted and returned to non-responsive. However, after 15 min hypergravity exposure, all genes became either non-responsive or even downregulated, indicating that the hypergravity effect is transient and that differential gene expression levels are regulated back to normal or are counter-regulated by downregulation (Figure 7).

A second pool of genes reacts with a delay. These genes are non-responsive after 20 s of hypergravity and react first after 75 s with an up- or downregulation. Almost all of the downregulated genes are non-responsive after 15 min of hypergravity. The genes that are upregulated after 75 s become either non-responsive after 15 min or become downregulated. This means that also this second, slightly delayed gene pool displays a transient differential gene expression response to hypergravity. Furthermore, a large fraction of the genes being non-responsive after 20 s of hypergravity remain non-responsive after 75 s and 15 min of hypergravity exposure (Figure 7). For control purposes, we performed the same analyses on microarray-only data (Supplementary Figure S4) and on the combined microarray and RNA-Seq data (Figure 7) and obtained very similar results.

Encouraged by these results, we included two further publicly available data sets from the NASA GeneLab in our analysis and compared the genome-wide transcriptomics data sets of immune cells from five different platforms: (i) parabolic flight, (ii) suborbital ballistic rocket flight, (iii) ground-based facility centrifuge, (iv) ground-based facility simulated microgravity, and (v) the International Space Station. While data sets i–iii were generated from human Jurkat T cells, set iv was generated from TK6 cells and set v from human T cells (Table 1). We analyzed which transcripts were differentially expressed in all data sets for all time points. Interestingly, we rediscovered the vacuolar H+-ATPase (V-ATPase) ATP6V1 as differentially expressed, a gene that we could already identify as gravity-regulated in a previous study [18]. This enzyme contains two multi-subunit domains: (1) V0, which is membrane-embedded, and (2) V1, which is associated with the cytosolic part of V0 [65,66]. V1 is composed of eight subunits (A-H, Figure 10). Subunit A is involved in the catalytic ATP-binding sites, whereas subunit D participates in the central stalk. The V-ATPase is known for its function as a H+ pump and in the pH regulation of intracellular compartments and is involved in enzyme activity, the dissociation of ligands from receptors, and the coupled transport of substrates across membranes [67,68,69,70]. Recently, it has been shown that the V-ATPase is also involved in additional cellular processes, e.g., as an anchorage site for the cytoskeleton, and plays an important role in cytoskeletal tethering [71]. Further studies described the association of the V-ATPase with the cytoskeleton and hypothesized that it is involved, among others, in the regulation of the cytoplasmic G-actin pools and in the crosslinking and stabilization of actin in filaments [72,73,74]. In this study, we compared the differential expression of the ATP6V1 subunits A and D between the different data sets (Figure 10). In our microarray analyses, we identified an upregulation during the short-term hypergravity phases of a parabolic flight (20 s) as well as of a suborbital ballistic rocket flight (75 s). These data could be validated by RT-qPCR experiments [18]. However, after 15 min of hypergravity exposure, both subunits could be identified as downregulated by microarray as well as by RNA-Seq analyses (Figure 10). This result is supported by the T cell ISS study from Chang and colleagues [43]. The analysis of the microarray data set available at GeneLab (GLDS-13) revealed a significant downregulation of ATP6V1A and ATP6V1D after 3 days of microgravity exposure and 1.5 h of T cell activation (Figure 7). In contrast, ATP6V1A and D were not significantly altered in the 48 h simulated microgravity study of TK6 lymphoblastoid cells from Chowdhury and colleagues [44]. We therefore conclude that the short-term up- and long-term downregulation of these transcripts is T-cell-specific. Interestingly, long-term space-related cytoskeletal degradation has been reported for Jurkat T cells previously [75,76]. Taken together, based on our combined multiple-platform and -technique meta-study, we could show the time-resolved differential expression of the ATPase subunits ATP6V1A and ATP6V1D from 20 s until days of altered gravity exposure. We hypothesize that long-term altered gravity exposure leads to decreased subunit expression, affecting the cytoskeletal status of T cells.

We further screened our 15 min hypergravity microarray and RNA-Seq data sets for differentially regulated transcripts. We identified nine Cluster of Differentiation (CD) molecules, CD1E, CD2AP, CD46, CD47, CD53, CD69, CD96, CD164, and CD226, as significantly differentially expressed (Figure 10). The comparison with the previously generated microarray data sets 23rd DLR PFC hypg and TEXUS-51 hypg showed the reverse effect. All transcripts were upregulated after 20 s and 75 s of hypergravity and significantly downregulated after 15 min of hypergravity exposure. Interestingly, the gene for the transmembrane protein CD47 showed the highest upregulation after 75 s, followed by a significant downregulation after 15 min hypergravity. CD47 is an anti-phagocytic receptor with a multitude of signaling functions including calcium signaling (reviewed in [77]). Calcium signaling plays an important role in altered gravity and has been described for various cell types (reviewed in [78]). However, no significant differential expression could be identified for CD47 in the T cell ISS data set GLDS-13. We therefore assume that the differential gene expression of CD47 is altered only for a limited period of time to support cellular calcium signaling and returns back to normal values.

Furthermore, the gene expression changes observed for CD96 were very interesting. During short-term hypergravity conditions up to 75 s, the gene expression was upregulated, and, after 15 min, it was downregulated. CD96 is an Ig superfamily member with three Ig-like domains and has been described as a T-cell-specific receptor that is upregulated upon T cell activation. It is involved in adhesive interactions of activated T cells during the late phase of the immune response (reviewed in [79]). Our analysis shows that the T cell surface molecule CD96 is first upregulated to support T cell activation, and, after 15 min of hypergravity exposure, it is downregulated again. The analysis of the long-term T cell ISS data set GLDS-13 revealed that CD96 expression is significantly downregulated after 1.5 h of microgravity exposure under ConA/CD28 activation [43]. Based on our data sets, we are not able to deduce if this downregulation is a continuous gravi-sensitive effect that leads to continuous downregulation that starts at least at minute 15, or if the dynamics are different between hyper- and microgravity. Interestingly, CD96 shares sequence similarities with CD226, which we also identified as differentially regulated in the same manner as CD96 in our data set comparison.

One transcript that was clearly altered in all data sets was CD69. CD69 is a calcium-dependent, type II lectin receptor and one of the earliest cell surface markers expressed by T cells following activation [43,80,81]. It is also expressed by immature thymocytes, B cells, natural killer (NK) cells, monocytes, neutrophils, and eosinophils, and is constitutively expressed by mature thymocytes and platelets [45]. Similarly to the other CD molecules, CD69 showed upregulated expression after 20 s and 75 s of hypergravity, followed by downregulation after 15 min of hypergravity. The long-term study GLDS-13 (Figure 10) displayed a significant downregulation of the CD69 transcripts. The GLDS-91 data set showed slight upregulation. We could not discriminate if this was an effect due to different exposure lengths, due to the different cell types, or due to simulated microgravity vs. real microgravity. The overall dynamics are in line with previous findings, where decreased expression of CD69 was reported, providing further evidence that T cell activation is significantly inhibited in altered gravity [43]. Expression of CD genes is not constant. Therefore, the effects of altered gravity on CD genes interferes with the time course of gene expression after T cell activation [43]. Gravity-related effects have not only been reported on the gene level, but also for the amount of CD69 membrane protein. Here, reduced CD69 levels on the surfaces of T cells within the first few hours of activation in zero gravity are proposed to interfere with the early immune response [80].

Taken together, all three CD molecules, CD47, CD69, and CD96, were demonstrated to be gravity-sensitive genes. As key players in the T cell immune response, they resemble different states of T cell activation, starting with Ca^2+^ signaling for CD47, the early T cell response for CD69, and the late T cell response for CD96. Therefore, these three genes are good potential targets for future studies that could potentially shed light on impaired functions of the immune system during spaceflight.

We were able to show in our study that transcriptomic data sets from databases such as GeneLab can be compared well with each other. Due to the highly complex process of generating altered gravity, comparative studies will likely include both RNA-Seq and microarray data sets. Here, we could demonstrate that, despite some weaknesses for each technology, studies can highly benefit from including both types of platforms. Particularly good agreement could be found when looking at pathway analyses. However, particularly strong effects could also be identified at the level of individual genes, as shown for ATP6V1 and CD surface molecules. Hypergravity phases in flight experiments are probably subject to further external factors such as vibrations, which cannot be controlled completely. Thus, experiments under standardized laboratory conditions allow the best possible degree of control and reproducibility. At the same time, the chosen measurement time of 15 min allowed a comparison with shorter hypergravity phases [10,11,12] derived from the parabolic flight and suborbital ballistic rocket experiments. In this comparison, possible adaptation processes could be identified, which are known from the microgravity environment [20].

In summary, in this study, we could increase our understanding of the transcriptional effects that are caused by short-term hypergravity, which adds further evidence to previous findings from our group and other groups (overview in Table 3). We could demonstrate that the transcriptional response to hypergravity is transient and shows a complex counter-response between 75 s and 15 min. The results add a further time point that could be measured on two different technologies which cross-validates transcriptional effects. Further, the counter-response could also be observed on the level of gene sets. Finally, further evidence of the involvement of the vacuolar H+ ATPase ATP6V could be generated and, additionally, the group of CD molecules was discovered that also showed strong involvement both on the short-term and long-term scale.

Using external data sets as a complement to one’s own data can highly increase the reproducibility and generalizability of the results found. Therefore, comparative studies will become more important as more genome-wide transcriptome studies are uploaded to the databases. Due to the enormous number of individual studies, the empirically evidenced comparability of methods, models, and platforms can only generate the knowledge gain that leads to a fundamental understanding and goes beyond individual findings. Using the example of adaptation to hypergravity analyzed here, it could be shown that hypergravity also represents the possibility to identify basic biological reactions to altered gravity.

## 4. Materials and Methods

All microgravity and hypergravity experiments were performed with Jurkat T cells. The term microgravity describes the physical condition of nearly weightlessness, whereby hypergravity is defined as a condition where the gravitational force is higher than on Earth. During a parabolic maneuver (either onboard a parabolic aircraft flight or a suborbital ballistic rocket), an object is weightless, flying on a Keplerian trajectory, described as an unpropelled body in ideally frictionless space subjected to a centrally symmetric gravitational field. During this free-fall trajectory, the result of all forces acting on the object other than gravity is nulled.

### 4.1. Preparation of Biological Specimen

The Jurkat T cells (ATCC Manassas, USA, Clone E6-1, TIB152™) were cultured in RPMI 1640 (Biochrom, Berlin, Germany Cat. Nr FG1215) medium, supplemented with 10% FCS and 1% Pen/Strep. During overnight (8 h+) incubation at 36.5 ± 0.5 °C, the Jurkat T cells were sedimented. To increase the cell concentration without centrifugation, the supernatant was removed. The Jurkat T cell concentration was adjusted to 5 × 10^6^ cells/mL. Then, 1 mL cells were aspirated into sterile, prewarmed (36.5 °C) 2 mL pipettes. The tips of the pipettes were closed with sterile silicone plugs, while the tops remained open. Pipettes containing cells were treated with either 15 min centrifugation at 9× *g* (hypg15) or 15 min incubation without centrifugation (Ctrl), at 36.5 °C. Here, 9× *g* centrifugation was carried out on a custom-built 9× *g* pipette centrifuge provided by KEK (Bad Schmiedeberg, Germany). The pipette centrifuge is designed so that the cells are not collected as pellets at the bottom, but along the entire pipette wall. For robustness, 10 samples per condition were generated, and a single centrifuge run consisted of 4 samples. In the next step, the pipettes were drained into 5 mL sterile, RNAse/DNase-free plastic tubes. In order to extract RNA, the 5 mL tubes contained TRIzol (Invitrogen, Carlsbad, CA, USA). In the second step, remaining cells were detached. Therefore, 1 mL of fresh TRIzol was aspirated and mixed with the solution by rolling the pipettes for 1 min followed by 5 aspirate/dispense steps. Ensuring RNA stability, the samples were incubated on ice before RNA extraction (QIAGEN RNeasy Mini Kit) was executed. Cells were homogenized by aspirating 5× into a syringe with a needle (B Braun, Melsungen, Germany, 0.8 × 80 mm) and released again. Per 1 mL of cell homogenate, 0.12 mL chloroform was added and the suspension was vortexed for 15 s. After incubation at RT for 5 min, samples were centrifuged (11,000× *g* at 4 °C for 15 min). The upper, aqueous phase was transferred into a clean 50 mL tube. Then, 0.6 mL RLT puffer including 1% 2-Mercaptoethanol was added. These samples were further processed according to the RNEasy protocol (QIAGEN, Hilden, Germany). The remaining RNA eluents were frozen at −150 °C.

### 4.2. Transcriptomics of Biological Specimen

RNA-Seq: Biological replicates were sequenced at the Core Facility for High-Throughput Genetics and Genomics, located at the Medical Faculty, Westfälische Wilhelms-Universität Münster. For all samples, the RNA integrity number (RIN) was measured. The four samples per condition with the highest RIN were selected. Poly-A-enriched, strand-specific antisense RNA sequences were applied on 1000 ng RNA that had been PCR-amplified for 8 cycles. The abovementioned samples were sequenced at a read depth of 25 × 106, involving 75 cycles. Demultiplexed fasta files were further analyzed at UZH.

Microarray: The fragmented and biotinylated RNA samples were prepared according to the standard Affymetrix WT PLUS Reagent Kit protocol (Affymetrix GeneChip^®^ WT PLUS Reagent Kit, 902280) from 100 ng total RNA starting material and 5.5 μg cDNA intermediate product. DNA targets were hybridized for 17 h at 45 °C on GeneChip Human Transcriptome Arrays 2.0. GeneChips were washed and stained in the Affymetrix Fluidics Station 450 according to the standard GeneChip Expression Wash, Stain and Scan protocol (Affymetrix GeneChip Wash, Stain and Scan Kit, 900720). Subsequently, the GeneChips were scanned using the Affymetrix 3000 7 G scanner. Raw CEL files were further analyzed at UZH.

### 4.3. RNA-Seq Sample Analysis

In the first step, the adapters of the demultiplexed fastq files were trimmed and their quality was assessed using TrimGalore version 0.6.5. The filtered files were aligned to the human genome (Homo_sapiens.GRCh38.99.gtf) via STAR version 2.7.3a. For standard quality control, FastQC version 0.11.9 and MultiQC version 1.9 were used. To obtain count matrices from aligned reads in bam files, the featureCounts package version 2.0.1 was applied with the appropriate command line arguments for strand specificity (reversely stranded, parameter “−s 2”) and for paired-end reads (“−*p*” argument). To detect differential expression, DESeq2 version 1.28.1 was used.

### 4.4. Microarray Sample Analysis

Raw CEL files were imported and RMA-normalized with the Bioconductor version 3.13 oligo package. Raw and processed quality control reports were generated with the ArrayQualityMetrics Bioconductor package. Array probesets and transcript clusters were annotated with manufacturer-specific annotation tables, supplemented by Ensembl biomaRt data (January 2021). With the help of the Bioconductor version 3.13 limma package, the log2 fold changes and *p*-values were calculated based on a linear regression model.

### 4.5. Previous and External Data Sets

Differential gene expression data from the external experiments 23rd DLR Parabolic Flight Campaign (PFC) hypg-1gIF and TEXUS-51 hypg-1gGC [18] were included in the study. For these, raw CEL data were processed as described for the internal data from this study. For the NASA GeneLab RNA-Seq data set GLDS-91, processed differential gene expression data including fold changes and *p*-values were acquired from GeneLab. For GLDS-13, the U133 Plus 2.0 data set, RMA-normalized signal intensity values were gathered from the NASA GeneLab database. Based on these, log2 fold changes and *p*-values were calculated.

### 4.6. Transcriptional Dynamics Analysis

Transcripts were classified as up- or downregulated according to their log fold change (logFC). The analysis was performed on the whole data set to characterize the overall logFC distribution. Further, only significant genes (adjusted *p*-value < 0.05) were considered. This allowed us to classify the set of transcripts into significantly upregulated transcripts (adj. *p*-value < 0.05 and logFC > 0), significantly downregulated transcripts (adj. *p*-value < 0.05, logFC < 0), and not significantly changed (adj. *p*-value > 0.05).

### 4.7. Upset Plots

Upset plots are an alternative to Venn diagrams. The different sets are plotted as bars, which represent their size. A line connecting two dots represents a set intersection. Dots represent a single set. A benefit of using upset plots is the fact that they can show contradictory results in the data. It is, for example, possible to plot if, for matched transcripts, both report an upregulated logFC or if the values do not agree, which would not be possible with Venn diagrams.

### 4.8. Set Inclusion Analysis

A hard cutoff of the adj. *p*-value at 0.05 might not always be desired. To achieve more transient results, the adj. *p*-value of one technology is fixed at 0.05 and the adj. *p*-value of the second technology is gradually lowered (from 0.05 to 0.0031). In the first iteration, both adj. *p*-values are fixed at 0.05. In the second iteration, the *p*-value of one technology is halved to 0.025 and so on. The count of significant genes thus decreases for the latter one. Count values of each set were transformed to frequencies.

### 4.9. Spearman Correlation Coefficient

To increase comparability between the two data sets, the Spearman rank correlation coefficient was determined. The coefficient can take on values between −1 and 1. A value of −1 implies perfect anticorrelation, 0 no correlation, and 1 perfect correlation. Correlation between the logFC of the two data sets was assessed as it was of interest to note if the two technologies reported similar logFC. The measure was computed for the whole data set and for the filtered data set (adj. *p*-value < 0.05). The results were plotted as 2D heat maps.

### 4.10. Differential Exon Usage (DEU)

For the RNA-Seq data set, DEXSeq version 1.34.1 was used. Bam files were counted via DEXSeq to generate exon alignment tables. A gff file was prepared from Homo_sapiens.GRCh38.99.gtf with dexseq_prepare_annotation2.py. Subsequently, exon counting was performed via dexseq_count.py, accounting for paired-end reads, reversely stranded library, and position-sorted input bam (arguments −*p* yes −s reverse −r pos −f bam). To investigate hypergravity-induced changes in exon usage, “design = sample + exon + condition: exon” was used as a design formula for contrast formation. DEU significance was calculated at the gene level. Genes with FDR-corrected *p*-value below 0.05 were considered differentially spliced/DEU.

For the microarray data set, raw CEL files were RMA-normalized and analyzed for differential exon usage in the Affymetrix Transcriptome Analysis Console (TAC) 4.0.3. This analysis fully leveraged the junction probes from the array to detect splicing. DEU significance was calculated at the gene level. Genes with FDR-corrected *p*-value below 0.05 were considered differentially spliced/DEU.

### 4.11. Chromosomal Mapping

DEGs were mapped to harboring chromosomes using Ensembl biomaRt (January 2021) annotations of gene location. Resulting distributions were analyzed using two independent Fisher exact tests per chromosome. The first was intended to investigate patterns in the total DEG distribution per chromosome. The second test assessed the ratio of up-/downregulated genes per chromosome. Resulting *p*-values were FDR-adjusted. Results below 0.05 were considered significant, below 0.01 highly significant. Expected numbers of DEGs per chromosome (assuming random distribution) were calculated by multiplying the total number of DEGs (all chromosomes) with the ratio of observable (adj. *p*-value not NA) genes on the respective chromosome over the total number of observable genes. Expected counts of upregulated DEGs were computed by multiplying the observed DEG count per chromosome with the global ratio between up- and downregulated genes.

### 4.12. Gene Set Enrichment

Fast pre-ranked gene set enrichment analysis (FGSEA, biomaRt version 3.13) was used to investigate enrichment of DEGs in gene ontology gene sets, obtained from the molecular signature database (MSigDB https://www.gsea-msigdb.org/gsea/downloads.jsp, accessed on 1 March 2021). Normalized enrichment scores (NES) were calculated based on the stat parameter (RNA-Seq) or the t test parameter (microarray), as recommended. Gene sets were considered enriched if they had an FDR-corrected *p*-value below 0.05.

### 4.13. Statistics

If not indicated differently, Benjamini–Hochberg false discovery rate-adjusted (FDR) *p*-values were used at cutoff 0.05 for significance. For analysis of randomness of distribution, Fisher’s exact test was used. *p*-values were FDR-adjusted.

## Figures and Tables

**Table 1:** Internal and external hyper- and microgravity data sets from different platforms measured by different transcriptomics techniques that were used for cross-correlation analysis of selected highly validated groups.

Experiment	Cell Type	Platform	Altered Gravity	Exposure Time	Transcriptomics
Hypg15_microarray	Jurkat T cell	Pipette centrifuge	Hyper-gravity 9× *g*	15 min	HTA2.0 microarray
Hypg15_RNA-Seq	Jurkat T cell	Pipette centrifuge	Hyper-gravity 9× *g*	15 min	Paired end poly-A RNA-Seq
23rd DLR PFC [18]	Jurkat T cell	Parabolic flight	Hyper-gravity 1.8× *g*	20 s	HTA2.0 microarray
TEXUS-51 [18]	Jurkat T cell	Sounding rocket	Hyper-gravity ~9× *g*	75 s	HTA2.0 microarray
GLDS-13 [43]	Activated primary human T cells	ISS, reference centrifuge	Micro-gravity, 1× *g*	3 days µg, activation in µg and 1× *g* for 1.5 h	Human U133 Plus 2.0
GLDS-91 [44]	TK6 lymphoblastoid cells	HARV Rotary Cell Culture System	Simulated micro-gravity	48 h	Paired-end total RNA-Seq

**Table 2:** List of significantly differentially expressed CD markers. Gene name, chromosomal localization, subcellular localization of the final protein, functional description of the gene, and presence in T cells (“+”: present, “-”: absent, “?”: ambiguous) are given.

Gene Name	Chromosome Location [50]	UniProt Subcellular Localization [51]	Human Gene Database Summary, Abbreviated [52]	T Cell Bearing [53]
CD53 molecule	1p13.3	Cell membrane, cell junction	Transmembrane 4 superfamily, signal transduction events that play a role in the regulation of cell development, activation, growth, and motility. Cell surface glycoprotein that is known to complex with integrins. It contributes to the transduction of CD2-generated signals in T cells and natural killer cells and has been suggested to play a role in growth regulation.	+
CD1E molecule	1q23.1	Golgi apparatus membrane, early and late endosome	Transmembrane glycoproteins, T cell surface glycoprotein CD1e, soluble, binds diacetylated lipids, including phosphatidyl inositides and diacylated sulfoglycolipids, and is required for the presentation of glycolipid antigens on the cell surface.	?
CD96 molecule	3q13.13	Membranes	Immunoglobulin superfamily type I membrane protein with role in the adhesive interactions of activated T cells during the late phase of the immune response.	+
CD47 molecule	3q13.12	Membranes	Membrane protein, involved in the increase in intracellular calcium concentration that occurs upon cell adhesion to extracellular matrix.	+
CD46 molecule	1q32.2	Membranes	Type I membrane protein, regulatory part of the complement system, cofactor activity for inactivation of complement components C3b and C4b by serum factor I, protects the host cell from damage by complement.	+
CD226 molecule	18q22.2	Cell membrane	Glycoprotein expressed on the surface of a subset of T cells. Involved in intercellular adhesion, lymphocyte signaling.	+
CD164 molecule	6q21	Membranes	Encodes a transmembrane sialomucin and cell adhesion molecule that regulates the proliferation, adhesion, and migration of hematopoietic progenitor cells.	+
CD2AP (associated protein)	6p12.3	Membrane ruffle, Cell junction, Cytoskeleton	Directly interacts with filamentous actin and a variety of cell membrane proteins through multiple actin binding sites, SH3 domains, proline-rich region containing binding sites for SH3 domains, adapter protein between membrane proteins, and the actin cytoskeleton.	+
CD69 molecule	12p13.31	Membranes	Calcium-dependent lectin superfamily of type II transmembrane receptors. Expression of the encoded protein is induced upon activation of T lymphocytes, may play a role in proliferation. Involved in lymphocyte proliferation and functions as a signal transmitting receptor in lymphocytes, natural killer (NK) cells, and platelets.	+

**Table 3:** Overview of novel insights from this work and how they add to the existing knowledge generated in previous studies.

	Previously Known	Novel Insights
Transcriptional response over time	Rapid adaptation, no continuous response [18]. Stable region 11p15.4 [19].	Complex dynamics with counter-response between 75 s and 15 min (Figure 5, Figure 6 and Figure 7)
Cross-validation of effects	Hyper- and microgravity cross-validation at time points 20 s, 75 s, 5 min [18].	15 min time point validation by independent transcriptomics analysis technologies (RNA microarray, RNA-Seq)
Gene set effects	GO “Histone binding function” enriched for TEXUS-54 µg [18]. GO “Transport”, “Cytosol”, “Nucleotide binding”, “Poly(A) RNA binding”, “Nuclear speck”, “RNA binding”, “Intracellular membrane-bound organelle”, “Regulation of alternative mRNA splicing via spliceosome” enriched for hyp-g in TEXUS-51 and GBF study [35]. GO “GPCR signaling”, “zing finger-involved DNA binding”, “bacterial defense”, “sensory perception genes” enriched in stable genes [19].	Counter-response between 20 s/75 s and 15 min (Figure 9)
ATP6V	Initially described: ATP6V1A significantly upregulated in hyp-g for TEXUS-51, ATP6V1D for 23rd DLR PFC [18].	Counter-response between 20 s/75 s and 15 min (Figure 10), long-term effects (Figure 10)
CD molecules	-	Strongly downregulated after 15 min, counter-response and long-term effects (Figure 10)

## Data Availability

The data sets generated during and analyzed during the current study are available in the GEO (Gene Expression Omnibus) repository (www.ncbi.nlm.nih.gov/projects/geo, accessed on 3 August 2021), accession no. GSE175800.

## Supplementary Materials

Supplementary materials can be found at https://www.mdpi.com/article/10.3390/ijms22168451/s1. References [38,39,40,41,82,83,84,85,86,87,88,89,90] are cited in Supplementary Materials.

## Abbreviations

FDR	False Discovery Rate
DEG	Differential Gene Expression
logFC	log2 fold change
DEU	Differential Exon Usage
NES	Normalized Enrichment Score
CD	Cluster of Differentiation protein
