# Supplementary material for: Rapid Transient Transcriptional Adaptation to Hypergravity in Jurkat T Cells Revealed by Comparative Analysis of Microarray and RNA-Seq Data
Source: Int J Mol Sci. 2021 Aug 6;22(16):8451. doi: 10.3390/ijms22168451 (PMC8395121; doi:10.3390/ijms22168451)
Supplement: Supplementary file 1 [file ijms-22-08451-s001.zip › ijms-1263036-supplementary.pdf]

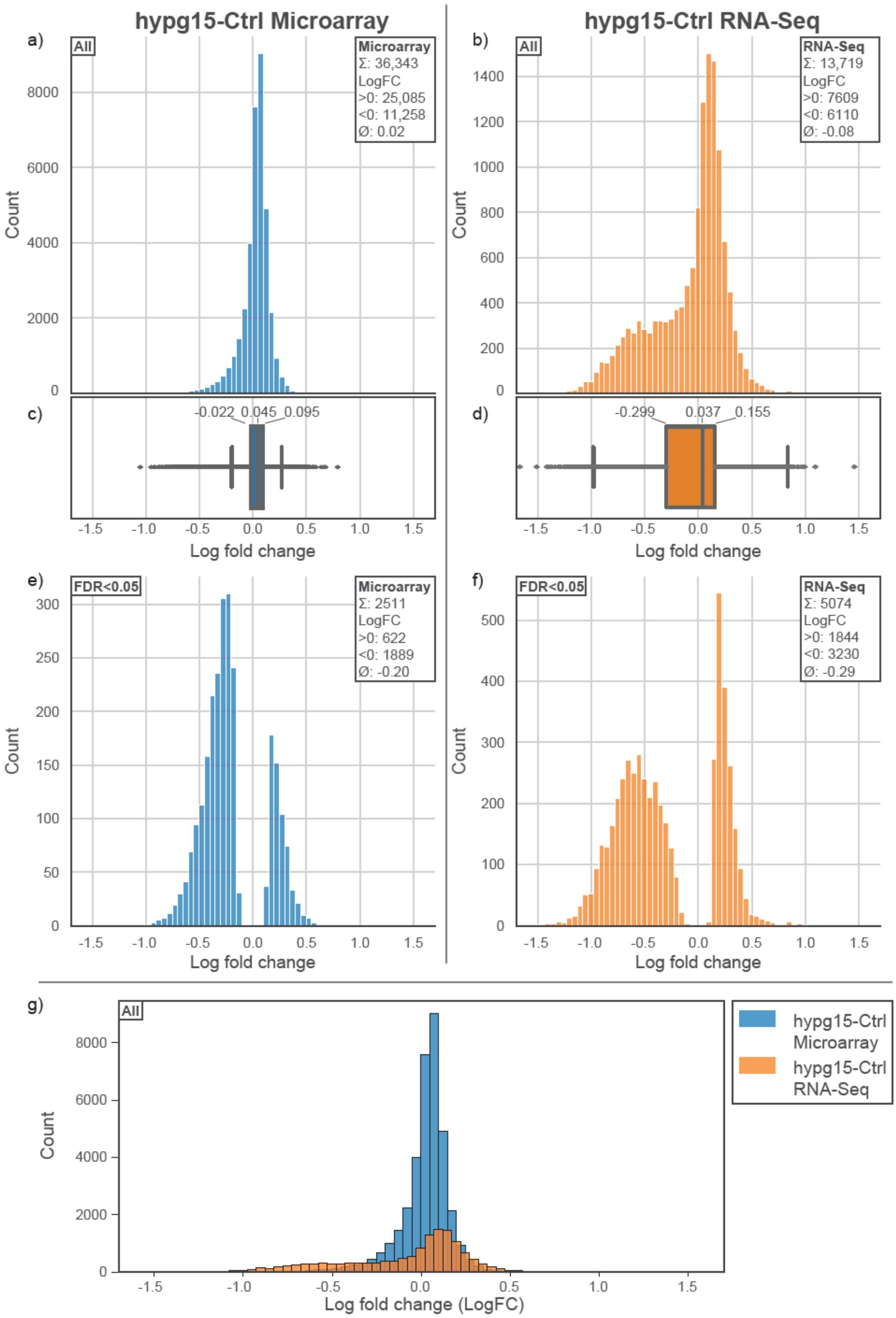

Figure S1. Overall distribution of differential gene expression for microarray and RNA-Seq data. Data is based on the same RNA samples for both technologies. **(a&b)** Separate histograms of each platform, binned number of genes per logarithmic fold change (log fold change, logFC). Log fold change scale is identical for both distributions, count scale is different. Key parameters are given in boxes: Count of all genes (Σ), Count of genes with fold change above 0 (>0) and below 0 (<0), and average fold change (Ø). **(c&d)** Structure of fold change distribution for all genes as boxplot. Box lines indicate 25%/50%/75% quantiles, for which the values are annotated. **(e&f)** Distribution of fold changes for both data sets, filtered for significantly differentially expressed genes. Key parameters are given in boxes. **(g)** Overlap histogram of both distribution for all genes reported by each platform, by logarithmic fold change.

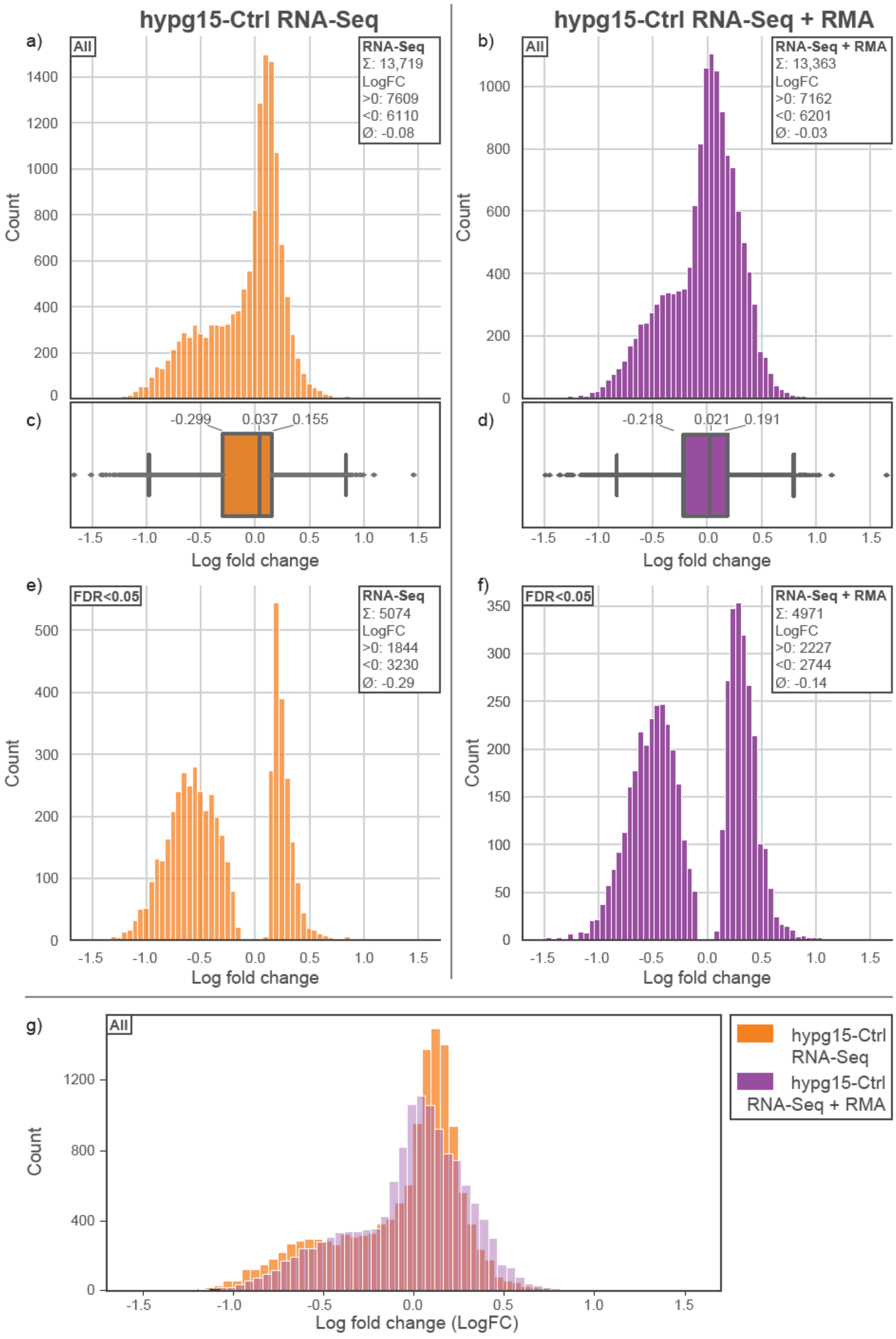

Figure S2. Visualization of the effects of quantile normalization. Overall distribution of differential gene expression for RNA-Seq data and post-counting quantile-normalized RNA-Seq data. **(a&b)** Separate histograms of each platform, binned number of genes by logarithmic fold change (log fold change, LFC). Log fold change scale is identical for both distributions, count scale is different. Key parameters are given in boxes: Count of all genes (Σ), Count of genes with fold change above 0 (>0) and below 0 (<0), and average fold change (Ø). **(c&d)** Structure of fold change distribution for all genes as boxplot. Box lines indicate 25%/50%/75% quantiles, for which the values are annotated. **(e&f)** Distribution of fold changes for both data sets, filtered for significantly differentially expressed genes. Key parameters are given in boxes. **(g)** Overlapping histogram of all genes reported by each platform, by logarithmic fold change.

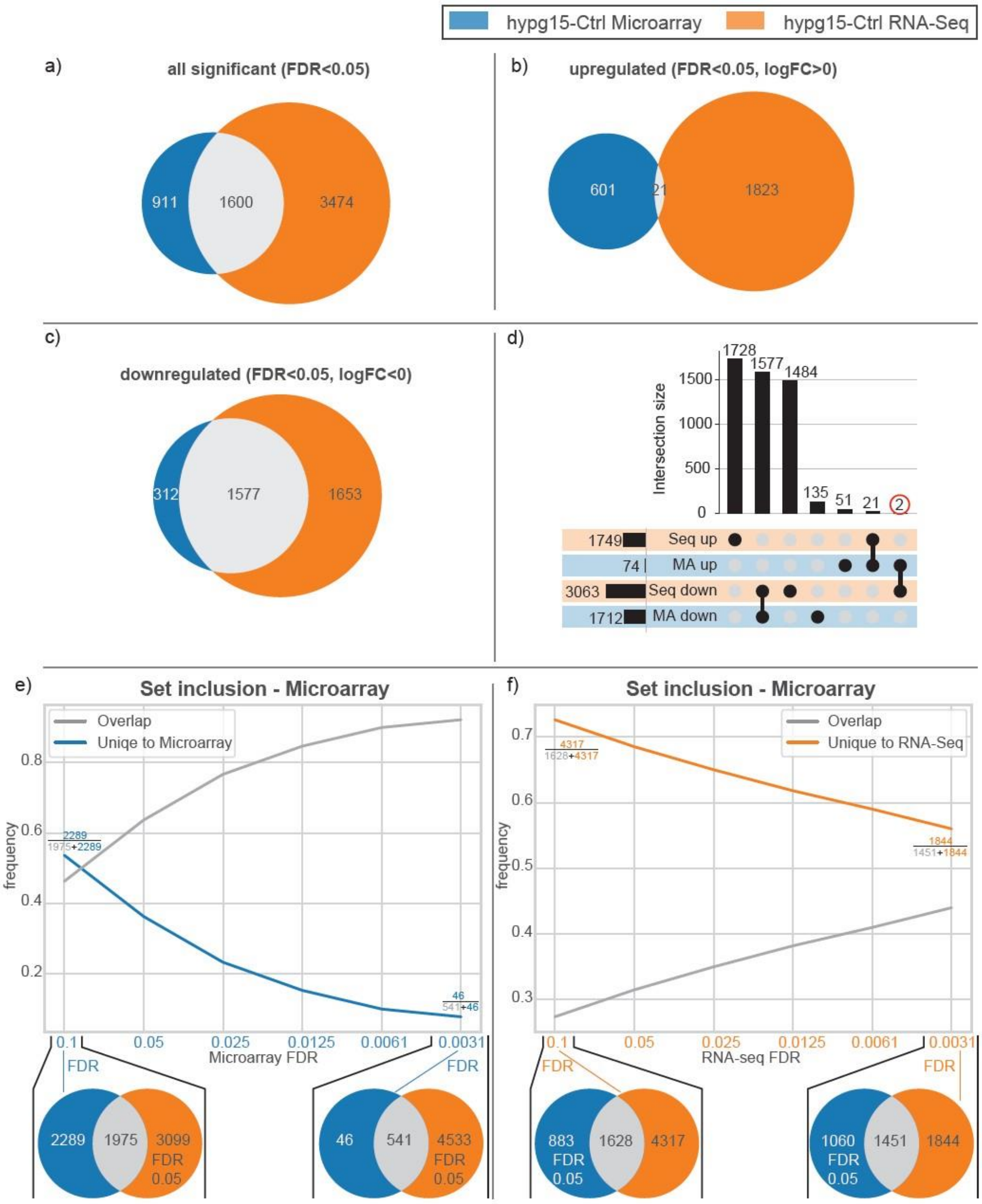

Figure S3. Consistency between technologies for differentially expressed genes. **(a)** Overlap of all significantly differentially expressed genes (FDR-adjusted p value below 0.05). **(b)** Overlap of significantly differentially expressed genes, only upregulated genes (logFC > 0). **(c)** Overlap of significantly differentially expressed genes, only downregulated genes (logFC < 0). **(d)** Upset plot of both data sets, separated by up- and downregulation. In contrast to a-c, only genes that were detected for both technologies were included (present on microarray, counted for RNA-Seq), leading to lower absolute numbers compared to a-c. Total numbers per set are reported on the left side (e.g. RNA-Seq up: 1749 genes are reported as significantly upregulated for RNA-Seq). Overlap with other sets is reported by black dots (e.g. 21 genes are significantly upregulated for RNA-Seq and microarray). Contradictory associations are reported, e.g. 2 genes were called upregulated for microarray and downregulated for RNA-Seq. **(e&f)** Set inclusion between both technologies. The relative overlap is reported between the two sets of significantly differentially expressed genes. The (FDR-adjusted) p value of one set is kept constant at 0.05 (g: RNA-Seq, h: microarray), while the other one is varied over the X axis, as visualized by the Venn diagrams at the bottom. This analysis compensates for effects where one gene is called significant for one technology and not for another if the p values are slightly below / slightly above 0.05. If all highly significant genes (lowest p value) are captured by the other technology at p below 0.05, this speaks for a high set inclusion. (MA: microarray, Seq: RNA-Seq).

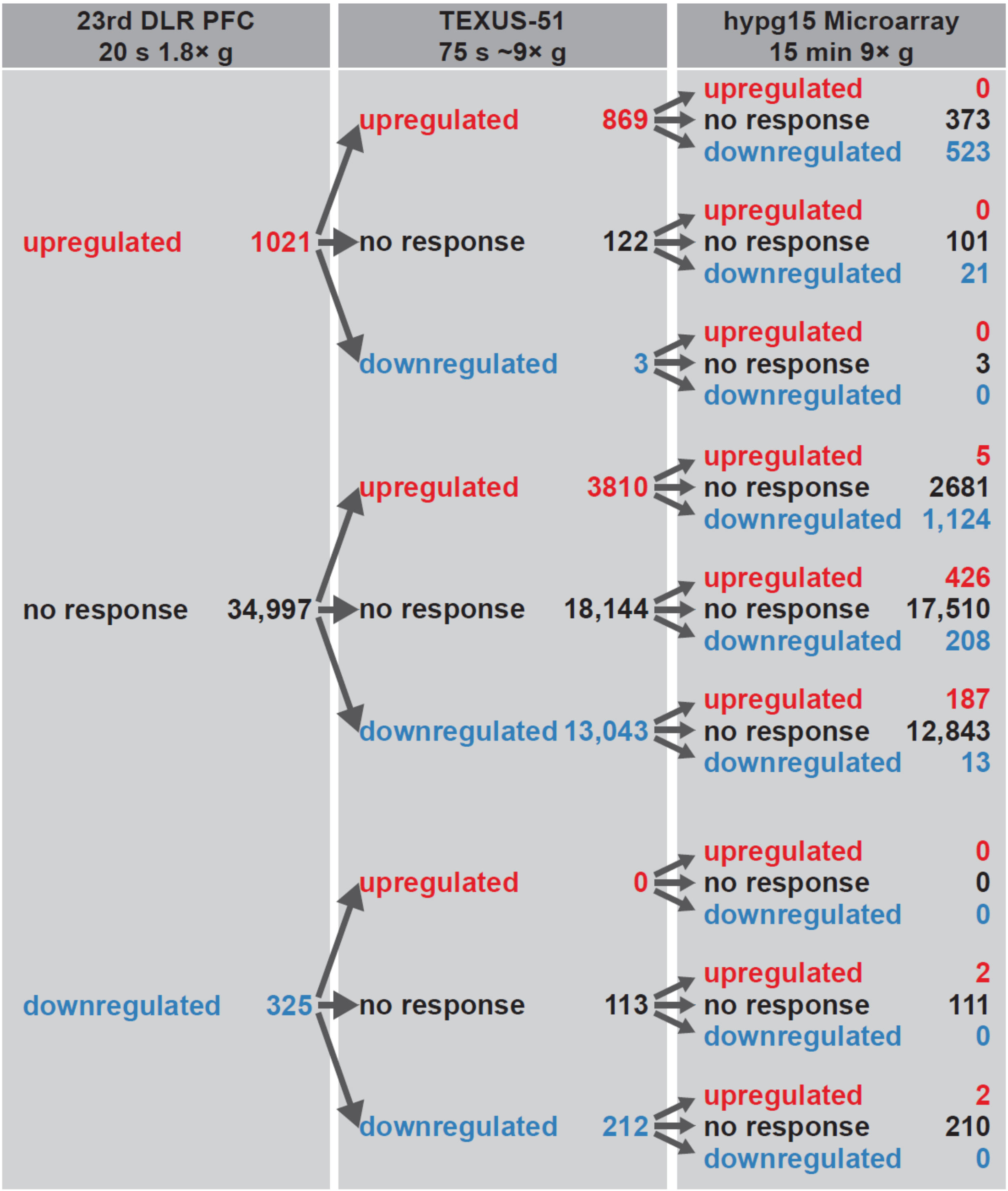

Figure S4. Corresponding differential expression between the 20s 1.8× g 23rd DLR PFC hypg vs 1g IF dataset, the 75s ~9× g TEXUS-51 hypg-1gGC dataset, and the 15 minutes hypg15 vs Ctrl dataset. Here, the overlap dataset was used for hypg15 (genes that were differentially expressed in both datasets). Additionally, all datasets were filtered for genes that were detected to be present by both technologies, resulting in fewer genes. For the first dataset, genes were separated into those that were significantly upregulated, downregulated, and not differentially expressed. Then, the genes in every group were split into those that were differentially upregulated, downregulated or not differentially expressed in the TEXUS-51 comparison, resulting in 9 categories. These 9 categories were consecutively split into 27 categories, depending on the behavior of the genes in the hypg15 vs Ctrl overlap dataset.

Table S1. 81 genes that show significant alternative splicing for the microarray (FDR<0.01) and the RNA-Seq (FDR<0.05) data set. These are the same genes as in figure 4d. The FDR values are listed for both sets.

Gene Symbol	Description	Microarray FDR	RNA-Seq FDR
ACAP2	ArfGAP with coiled-coil, ankyrin repeat and PH domains 2	0.004973537	0.022031017
ALG3	ALG3, alpha-1,3- mannosyltransferase	0.008679396	0.049381583
ANKRD11	ankyrin repeat domain 11	0.008679396	0.000921444
ATP2A3	ATPase, Ca++ transporting, ubiquitous	0.008679396	0.041537788
BNIP3L	BCL2/adenovirus E1B 19kDa interacting protein 3-like	0.006548074	0.00452815
BOD1L1	biorientation of chromosomes in cell division 1-like 1	0.007986108	0.005237986
C5orf24	chromosome 5 open reading frame 24	0.007986108	0.001947583
CAMK4	calcium/calmodulin-dependent protein kinase IV	0.007986108	0.000244331
CANX	calnexin	0.004973537	2.74E-05
CDC42SE2	CDC42 small effector 2	0.008679396	0.00050042
CDK1	cyclin-dependent kinase 1	0.004973537	0.02697497
CERS6	ceramide synthase 6	0.009717472	9.16E-07
CFL1	cofilin 1 (non-muscle)	0.007498465	0.03090761
CGGBP1	CGG triplet repeat binding protein 1	0.006548074	3.25E-06
CNBP	CCHC-type zinc finger, nucleic acid binding protein	0.008679396	8.66E-05
CPSF6	cleavage and polyadenylation specific factor 6	0.008679396	3.53E-12
DCTN4	dynactin 4 (p62)	0.007986108	0.023598109
DDX3X	DEAD (Asp-Glu-Ala-Asp) box helicase 3, X-linked	0.007498465	0.016783744
EIF3J	eukaryotic translation initiation factor 3, subunit J	0.008679396	0.027573981
FNBP1	formin binding protein 1	0.008679396	1.41E-06
GNAS	GNAS complex locus	0.006548074	0.025645083
HHIP	hedgehog interacting protein	0.007498465	0.001776847
HMGB1	high mobility group box 1	0.004579349	0.027802211
HNRNPH3	heterogeneous nuclear ribonucleoprotein H3 (2H9)	0.009133645	0.038144335
HUWE1	HECT, UBA and WWE domain containing 1, E3 ubiquitin protein ligase	0.007986108	0.037744929
ITGA4	integrin alpha 4	0.009133645	5.15E-05
KIAA1109	KIAA1109	0.004973537	0.009518614
LANCL1	LanC lantibiotic synthetase component C-like 1 (bacterial)	0.004973537	4.92E-05
LPP	LIM domain containing preferred translocation partner in lipoma	0.007498465	0.002956267
LRP6	LDL receptor related protein 6	0.004973537	0.017647784
LSAMP	limbic system-associated membrane protein	0.004973537	0.002084309
MBNL1	muscleblind like splicing regulator 1	0.004973537	0.023589531
MCM7	minichromosome maintenance complex component 7	0.007498465	0.031508684
MOB1A	MOB kinase activator 1A	0.006548074	0.000507735
MORF4L1	mortality factor 4 like 1	0.004973537	2.85E-05
NAA50	N(alpha)-acetyltransferase 50, NatE catalytic subunit	0.008679396	1.80E-05
NAP1L1	nucleosome assembly protein 1-like 1	0.008679396	0.023425271
NCBP2	nuclear cap binding protein subunit 2	0.004973537	0.000753888
NF1	neurofibromin 1	0.004973537	0.012868621
NUCB2	nucleobindin 2	0.004973537	0.025609005
NUP50	nucleoporin 50kDa	0.006548074	0.000370696
PAIP2	poly(A) binding protein interacting protein 2	0.004973537	0.001767831
PCF11	PCF11 cleavage and polyadenylation factor subunit	0.009717472	0.026723816
PCNP	PEST proteolytic signal containing nuclear protein	0.004973537	0.003945422
PELI2	pellino E3 ubiquitin protein ligase family member 2	0.009133645	0.017068938
PHIP	pleckstrin homology domain interacting protein	0.003279384	0.0006681
PNISR	PNN-interacting serine/arginine-rich protein	0.009717472	4.89E-10
PPP2R5E	protein phosphatase 2, regulatory subunit B, epsilon isoform	0.006548074	0.002619568
PRPF40A	PRP40 pre-mRNA processing factor 40 homolog A	0.004973537	1.88E-06
PSIP1	PC4 and SFRS1 interacting protein 1	0.004973537	4.90E-05
RB1	retinoblastoma 1	0.004973537	0.009647187
RBBP6	retinoblastoma binding protein 6	0.004973537	0.000311696
RBM15	RNA binding motif protein 15	0.009133645	5.80E-06
RBMX	RNA binding motif protein, X-linked	0.007498465	4.06E-06
RMND5A	required for meiotic nuclear division 5 homolog A	0.004973537	0.01301538
RPL23	ribosomal protein L23	0.004973537	0.005991864
RPL6	ribosomal protein L6	0.004973537	0.020125847
RPS6KB1	ribosomal protein S6 kinase, 70kDa, polypeptide 1	0.007498465	0.01772716
SCAI	suppressor of cancer cell invasion	0.007986108	3.41E-10
SH3GLB1	SH3-domain GRB2-like endophilin B1	0.008679396	0.0036433
SKP2	S-phase kinase-associated protein 2, E3 ubiquitin protein ligase	0.009133645	0.004390511
SMAD5	SMAD family member 5	0.009717472	0.000333737
SMIM13	small integral membrane protein 13	0.009717472	0.001225407
SRSF3	serine/arginine-rich splicing factor 3	0.004973537	4.36E-06
STAT1	signal transducer and activator of transcription 1	0.007986108	0.00736193
STT3B	STT3B, subunit of the oligosaccharyltransferase complex (catalytic)	0.009717472	0.031706556
STX6	syntaxin 6	0.004230313	0.003242792
SUB1	SUB1 homolog, transcriptional regulator	0.004973537	0.00037077
TBL1XR1	transducin (beta)-like 1 X-linked receptor 1	0.006548074	0.030942548
TCF12	transcription factor 12	0.008679396	0.014978197
TDP2	tyrosyl-DNA phosphodiesterase 2	0.006548074	0.03353481
TIA1	TIA1 cytotoxic granule-associated RNA binding protein	0.004973537	1.02E-05
TLK1	tousled-like kinase 1	0.004973537	0.013703281
TMX3	thioredoxin-related transmembrane protein 3	0.009133645	0.000339756
TXLNGY	taxilin gamma pseudogene, Y-linked	0.007498465	0.029529625
UBA2	ubiquitin-like modifier activating enzyme 2	0.007986108	0.022625803
UBE2K	ubiquitin conjugating enzyme E2K	0.006548074	4.54E-08
USP7	ubiquitin specific peptidase 7 (herpes virus-associated)	0.008679396	0.002118567
ZBTB44	zinc finger and BTB domain containing 44	0.004973537	0.000371103
ZNF107	zinc finger protein 107	0.004973537	0.000286793
ZNF83	zinc finger protein 83	0.008679396	0.017662585

Supplementary Results and Discussion. Extended comparison of RNA microarray versus RNA- Seq overall distribution and differential gene expression.

S1.1 Direct comparison of RNA microarray versus RNA-Seq

We wanted to understand the general performance of both technologies on our samples better. Therefore, an overall characterization of fold change distribution was performed. The distribution is displayed for the microarray and RNA-Seq analyses, including boxplots to describe the overall statistics (figure S1a-d). The microarray data set reported 36,434 genes while the RNA-Seq data set included only 13,719 genes. This is likely a consequence of the fixed design of the microarray which always reports all spotted genes, compared to the RNA-Seq analysis setup that requires a certain number of counts to label a gene as present. Figure S1a shows that the distribution of the microarray data is slightly shifted to the left, indicating an overrepresentation of the downregulated genes. This effect can be seen even more pronounced in the RNA-Seq data set in figure S1b. Here, strongly downregulated genes form a shoulder on the left side of the plot leading to a skew distribution of negative log fold changes. The upregulated gene fractions look similar for both analysis technologies. The statistical distribution of the microarray is shifted towards upregulation compared to RNA-Seq with the 25% quantile at logarithmic fold change (logFC) -0.022 (microarray) compared to -0.299 (RNA-Seq), the 50% quantile at 0.045 vs 0.037, but the 75% quantile at 0.095 vs 0.155. Generally, RNA-Seq seems to have a higher dynamic range in fold change distribution than the microarray: RNA-Seq has 159 genes with a fold change below -1 while only one gene could be identified for the microarray data set. Looking at fold changes above 0.5 RNA-Seq displayed 174 genes whereas only 19 genes could be found for the microarray data set. When analyzing the significantly differentially expressed genes, defined as false discovery rate-adjusted p value below 0.05 (FDR<0.05), the picture changed (figure S1e-f): The microarray data set was only able to detect 2511 significantly different genes, while RNA-Seq detected 5074 genes. Next, we analyzed the number of up- and downregulated genes for both assays. The microarray set only had 622 significant differentially expressed genes (DEGs) that were upregulated, in contrast to 1889 downregulated genes, which is a ratio of approximately 1:3. In contrast, RNA-Seq showed a more balanced behavior with 1844 upregulated genes and 3230 downregulated genes, which represents a ratio of more than 1:2. Generally, the trend for the microarray is more towards downregulation for the significant genes, which is contrarily to the overall data set which appeared shifted towards upregulation. Finally, the overall distributions of fold changes were plotted as an overlay to better visualize differences between the two data sets (figure S1g). The complete RNA-Seq data set and the significantly different genes (FDR<0.05) RNA-Seq data sets resembled two distinct normal distributions below and above logFC 0 (figure S1b,f). Surprisingly, for the microarray data set, this was not the case. The distribution seemed shifted and compressed and resembled only one normal distribution (figure S1a,e). Next to the lower dynamic range of microarrays, this effect could be caused by the microarray normalization method, robust multiarray averaging (RMA). One central step of RMA is a quantile normalization which expects overall equal distributions for all data sets and re-shapes the distribution of values. Due to the unbalanced distribution of fold changes, RMA could potentially disturb the skewed distribution by re-shaping it to become more homogenously distributed. To test whether this could be the case, quantile normalization was applied on the raw counts of the RNA-Seq data set prior to calculating fold changes (figure S2). The application did not fully resemble the distribution of the microarray but led to a compressed and partly homogenized fold change distribution with significantly less skew and an upwards-shifted boxplot. The limited effect of re-shaping the distribution was expected due to the fact that the RNA-Seq raw counts display already a highly processed state of the data set with less entries. However, there still was a pronounced shoulder for the RMA-normalized data set (figure S2 b), but the boxplot quantiles were more equal (25%: -0.218, 50%: 0.021, and 75%: 0.191, vs. 25%: -0.299, 50%: 0.037, and 75%: 0.155 for the original RNA-Seq set). Additionally, the number of significantly upregulated vs. downregulated genes became more balanced with 2227 upregulated and 2744 downregulated. Therefore, RMA could be one likely explanation why the overall skew is not conserved for the microarray data set.

As de-facto standard of normalization, there is no general solution to this problem. The current AffyMetrix-specific Transcriptome Analysis console (TAC) supports only RMA and (GC)RMA [1]. The quantile normalization part affects overall distribution of the statistics and smooths uneven distributions. This is because RMA and many other approaches assume most genes to be equally expressed in all conditions and that differentially expressed genes are symmetrically up- and downregulated [2, 3]. There are some proposed alternative algorithms that overcome these limitations,

including GRSN [4], HMM assisted normalization [5], CrossNorm [6], spike in control-based normalization [7] only to name a few (21 methods reviewed in [8]). For all of them, the underlying idea is to identify invariant features as a reference subset for normalization, in contrast to using the entire set as in the classical methods. This is either performed by automatic data-driven approaches, external negative controls, or all genes versus several references. Still, none of these algorithms is free of assumptions, they all bear similar problems as RMA [8]. On the other hand, the utilized DESeq2 normalization for RNA-Seq, shows excellent performance with unbalanced data sets with a large fraction of differentially expressed genes [9]. This is in line with our results. Additionally, RNA-Seq is also more sensitive to differential expression [10-13]. Still, the overall overlap between both data sets was in good agreement (figure S3). Especially RNA-Seq was able to detect most of the differentially expressed genes that appeared for the microarray data sets at strict FDR cutoffs (figure S3e). The microarray also detected many of the highly significant differentially expressed genes for the RNA-Seq data set (figure S3f). The correlation coefficient between fold changes of 0.68 did not indicate a perfect numerical match. However, the exact order of fold changes between differentially expressed genes is not most relevant for these datasets with thousands of differentially expressed genes. The high agreement between significantly differentially expressed genes and the almost identical findings on the aggregated level (figure 2) indicates large comparability.

S1.2. Microarray and RNA-Seq based DEGs were widely overlapping for downregulated but not for upregulated genes

After analyzing the general distribution of fold changes and differentially expressed genes (DEGs), the next step was to assess if the same DEGs could be found by both technologies (figure S3). Generally, RNA-Seq identified much more genes differentially expressed (5074) compared to the microarray analysis (2511). From the 2511 DEGs identified in the microarray-based study, 1600, i.e., over 60% of DEGs were also detected in the RNA-Seq data set (figure S3a). When differentiating by only upregulated and downregulated genes (figure 2b-c), the overlap for the downregulated genes was around 85% with 1577 overlapping DEGs but only 3% with 21 overlapping DEGs for the upregulated genes. The strong skew could be potentially explained by the upwards-shifted distribution for the microarray. Next, we wanted to understand if the aforementioned behavior potentially leads to contradictory results, e.g. upregulated gene as the result of one technology and downregulated gene for the other technology. An upset plot analysis was generated and filtered for genes that appeared in both data sets (figure S3d). The picture was comparable to the previous analysis: Downregulated genes were strongly shared between technologies, whereas for upregulated genes only a minority of 21 DEGs were overlapping. The set of contradictory results was very small with only two genes appearing as upregulated on the microarray and downregulated with RNA-Seq. However, contradictory results are possible in principle. The previous analyses were based on fixed FDR value cutoffs. This bears the problem that due to random noise, genes that are called significant for one technology with an FDR slightly below 0.05 could be called non-significant for the other with an FDR slightly above 0.05. To overcome this issue, a set inclusion analysis was performed: For the comparison of the DEG overlap between two data sets, the FDR cutoff for one data set was kept constant at 0.05 and the other was lowered stepwise (figure S3e-f). If at very low FDR for one data set, most DEGs of this data set were also contained in the larger set at FDR<0.05, this would indicate that highly significant genes for one data set were generally also detected for the other data set. If not, this would indicate that additional genes were found for the technology at very low FDR. For the microarray data set (figure S3e), at a low FDR of 0.0031, 541 or 92% of significant genes were contained in the RNA-Seq set at FDR<0.05. This indicated a large set inclusion and therefore a good agreement. On the other hand, RNA-Seq (figure S3f) only showed an overlap of 44% (1451 out of 3295) at p<0.0031 with the microarray set at FDR<0.05. The RNA-Seq set still contained more significant genes at this low FDR than the microarray set at FDR<0.05, an indicator of substantially higher statistical power of the RNA-Seq data set. Still, the most significant genes were also identified by the microarrays: when filtering for an extremely low p cutoff of FDR $<10^{-12}$ for the RNA-Seq data set, the overlap with the microarray data set at FDR<0.05 was 86% i.e., 467 significant genes for RNA-Seq (not shown in figure S3). Therefore, RNA-Seq had a substantially better ability to detect significantly differentially expressed genes, however the strongest DEGs were identified by both technologies.

References

1. AffyMetrix, Transcriptome Analysis Console 4.0.1 - User Guide. In 2019.
2. Lovén, J.; Orlando, D. A.; Sigova, A. A.; Lin, C. Y.; Rahl, P. B.; Burge, C. B.; Levens, D. L.; Lee, T. I.; Young, R. A., Revisiting Global Gene Expression Analysis. *Cell* **2012,** 151, (3), 476–482-476–482.
3. Robinson, M. D.; Oshlack, A., A scaling normalization method for differential expression analysis of RNA-seq data. *Genome Biology* **2010,** 11, (3), R25-R25.
4. Pelz, C. R.; Kulesz-Martin, M.; Bagby, G.; Sears, R. C., Global rank-invariant set normalization (GRSN) to reduce systematic distortions in microarray data. *BMC Bioinformatics* **2008,** 9, (1), 520-520.
5. Landfors, M.; Philip, P.; Rydén, P.; Stenberg, P., Normalization of high dimensional genomics data where the distribution of the altered variables is skewed. *PloS One* **2011,** 6, (11), e27942-e27942.
6. Cheng, L.; Lo, L.-Y.; Tang, N. L. S.; Wang, D.; Leung, K.-S., CrossNorm: a novel normalization strategy for microarray data in cancers. *Scientific Reports* **2016,** 6, 18898-18898.
7. Chen, K.; Hu, Z.; Xia, Z.; Zhao, D.; Li, W.; Tyler, J. K., The Overlooked Fact: Fundamental Need for Spike-In Control for Virtually All Genome-Wide Analyses. *Molecular and Cellular Biology* **2016,** 36, (5), 662–667-662–667.
8. Liu, X.; Li, N.; Liu, S.; Wang, J.; Zhang, N.; Zheng, X.; Leung, K.-S.; Cheng, L., Normalization Methods for the Analysis of Unbalanced Transcriptome Data: A Review. *Frontiers in Bioengineering and Biotechnology* **2019,** 7.
9. Evans, C.; Hardin, J.; Stoebel, D. M., Selecting between-sample RNA-Seq normalization methods from the perspective of their assumptions. *Briefings in Bioinformatics* **2017,** 19, (5), 776–792-776–792.
10. Kogenaru, S.; Qing, Y.; Guo, Y.; Wang, N., RNA-seq and microarray complement each other in transcriptome profiling. **2012,** 13, 629-629.
11. Mills, J. D.; Kawahara, Y.; Janitz, M., Strand-Specific RNA-Seq Provides Greater Resolution of Transcriptome Profiling. **2013,** 14, 173-181.
12. Zhang, W.; Yu, Y.; Hertwig, F.; Thierry-Mieg, J.; Zhang, W.; Thierry-Mieg, D.; Wang, J.; Furlanello, C.; Devanarayan, V.; Cheng, J.; Deng, Y.; Hero, B.; Hong, H.; Jia, M.; Li, L.; Lin, S. M.; Nikolsky, Y.; Oberthuer, A.; Qing, T.; Su, Z.; Volland, R.; Wang, C.; Wang, M. D.; Ai, J.; Albanese, D.; Asgharzadeh, S.; Avigad, S.; Bao, W.; Bessarabova, M.; Brilliant, M. H.; Brors, B.; Chierici, M.; Chu, T.-M.; Zhang, J.; Grundy, R. G.; He, M. M.; Hebbring, S.; Kaufman, H. L.; Lababidi, S.; Lancashire, L. J.; Li, Y.; Lu, X. X.; Luo, H.; Ma, X.; Ning, B.; Noguera, R.; Peifer, M.; Phan, J. H.; Roels, F.; Rosswog, C.; Shao, S.; Shen, J.; Theissen, J.; Tonini, G. P.; Vandesompele, J.; Wu, P.-Y.; Xiao, W.; Xu, J.; Xu, W.; Xuan, J.; Yang, Y.; Ye, Z.; Dong, Z.; Zhang, K. K.; Yin, Y.; Zhao, C.; Zheng, Y.; Wolfinger, R. D.; Shi, T.; Malkas, L. H.; Berthold, F.; Wang, J.; Tong, W.; Shi, L.; Peng, Z.; Fischer, M., Comparison of RNA-seq and microarray-based models for clinical endpoint prediction. *Genome Biology* **2015,** 16, (1), 133-133.
13. Zhao, S.; Fung-Leung, W.-P.; Bittner, A.; Ngo, K.; Liu, X., Comparison of RNA-Seq and Microarray in Transcriptome Profiling of Activated T Cells. **2014,** 9, e78644-e78644.
